# Broad spectrum of β-lactamase coverage and potent antimicrobial activity of xeruborbactam in combination with meropenem against carbapenemase-producing Enterobacterales, including strains resistant to new β-lactam/β-lactamase inhibitor combinations

**DOI:** 10.1128/aac.00533-25

**Published:** 2025-07-25

**Authors:** Lucía Sánchez-Peña, Salud Rodríguez-Pallares, Pablo Aja-Macaya, Tania Blanco-Martín, Lucía González-Pinto, Gloria Pérez-Rodríguez, Christophe Le Terrier, Inés Portillo-Calderón, Esther Recacha, Cristina Riazzo, Juan Carlos Vázquez-Ucha, Alejandro Beceiro, Belén Aracil, Jesús Oteo-Iglesias, Luis Martínez-Martínez, Laurent Poirel, Germán Bou, Jorge Arca-Suárez

**Affiliations:** 1 Servicio de Microbiología Clínica, Instituto de Investigación Biomédica A Coruña (INIBIC), Complexo Hospitalario Universitario A Coruña 16811, A Coruña, Spain; 2 CIBER de Enfermedades Infecciosas, CIBERINFEC, Instituto de Salud Carlos III 38176, Madrid, Spain; 3 Medical and Molecular Microbiology, Faculty of Science and Medicine, University of Fribourg 98839, Fribourg, Switzerland; 4 Division of Intensive Care, Department of Acute Care Medicine, Geneva University Hospitals 30578, Geneva, Switzerland; 5 Department of Anaesthesiology, Pharmacology, Intensive Care and Emergency Medicine, Faculty of Medicine, University of Geneva 28535, Geneva, Switzerland; 6 Laboratorio de Referencia para Tipado Molecular y Detección de Mecanismos de Resistencia a Antimicrobianos de Andalucía (PIRASOA), Unidad de Gestión Clínica de Microbiología y Enfermedades Infecciosas, Hospital Universitario Virgen Macarena. Instituto de Biomedicina de Sevilla (IBIS), CSIC, Universidad de Sevilla 16778, Seville, Spain; 7 Unidad de Microbiología, Hospital Universitario Reina Sofía e Instituto Maimónides de Investigación Biomédica de Córdoba (IMIBIC) 215147, Córdoba, Spain; 8 Laboratorio de Referencia e Investigación en Resistencia a Antibióticos e Infecciones Relacionadas con la Asistencia Sanitaria, Centro Nacional de Microbiología, Instituto de Salud Carlos III, Majadahonda 38176, Madrid, Spain; 9 Departamento de Química Agrícola, Edafología y Microbiología, Universidad de Córdoba 16735, Córdoba, Spain; 10 Swiss National Reference Center for Emerging Antibiotic Resistance (NARA), University of Fribourg 27211, Fribourg, Switzerland; 11 Departamento de Fisioterapia, Medicina y Ciencias Biomédicas, Universidad de A Coruña 16737 , A Coruña, Spain; Universita degli Studi di Roma "La Sapienza", Rome, Italy

**Keywords:** carbapenemase-producing Enterobacterales, Enterobacterales, carbapenemase, xeruborbactam, beta-lactamase inhibitor, beta-lactamase, beta-lactam

## Abstract

Xeruborbactam is a broad-spectrum boronate-type β-lactamase inhibitor. We aimed to evaluate its activity in combination with meropenem and compare it with other β-lactam/β-lactamase inhibitor combinations against Enterobacterales. The following isolates were screened: (i) an isogenic collection of 94 *Escherichia coli* isolates producing β-lactamases under wild-type and low-permeability conditions, (ii) 300 genetically diverse clinical Enterobacterales isolates producing the three main carbapenemase types (KPC-like, OXA-48-like, and metallo-β-lactamases), and (iii) two collections of isolates producing mechanisms of resistance to β-lactam/β-lactamase inhibitor combinations, such as KPC variants or PBP3 insertions combined with metallo-β-lactamases (MBLs). The MICs of meropenem, meropenem/xeruborbactam, meropenem/vaborbactam, imipenem, imipenem/relebactam, cefepime, cefepime/taniborbactam, ceftazidime, ceftazidime/avibactam, aztreonam, and aztreonam/avibactam were determined by reference broth microdilution and interpreted following the European Committee on Antimicrobial Susceptibility Testing guidelines, using the breakpoint of the β-lactam alone for not yet approved combinations. Resistance mechanisms of all clinical isolates were analyzed by whole genome sequencing. Meropenem/xeruborbactam had the broadest spectrum against the isogenic collection, although higher MICs were noted for transformants producing IMP-23, SPM-1, and NDM enzymes (these latter only when produced under low-permeability conditions). Meropenem/xeruborbactam displayed the most potent activity against the collection of 300 clinical strains (MIC_50/90_ ≤0.06/≤0.06 mg/L). Xeruborbactam restored meropenem activity against the strains carrying resistance mechanisms to β-lactam/β-lactamase inhibitor combinations, including strains producing KPC variants or MBLs in combination with additional chromosomal alterations (MIC range: ≤0.06–0.25 and ≤0.06–4 mg/L, respectively). Our findings highlight the potential of xeruborbactam in combination with meropenem as a promising treatment against carbapenemase-producing Enterobacterales, including strains with emerging resistance to other β-lactam/β-lactamase inhibitor combinations.

## INTRODUCTION

Infections caused by carbapenemase-producing Enterobacterales (CPE), classified as “critical priority pathogens” by the World Health Organization (WHO), pose an important healthcare threat (1). This is due to the very limited repertoire of active antibiotics available for combating infections caused by these pathogens, as carbapenemases may exhibit a wide spectrum of activity against all β-lactams, commonly resulting in the so-called difficult-to-treat phenotypes (2). This complex clinical scenario is further exacerbated by the growing prevalence of CPE in all geographical regions and particularly producers of metallo-β-lactamases (MBLs), such as NDM, VIM, and IMP-type enzymes, which are recalcitrant to most clinically available newly developed β-lactam/β-lactamase inhibitor combinations (3).

Thanks to intense drug discovery efforts in the last few decades, we are now witnessing a new era of β-lactam/β-lactamase inhibitor combinations with potent activity against multidrug-resistant Gram-negative microorganisms, including CPE (4). Most combinations usually rely on the partnership between broad-spectrum β-lactam antibiotics (e.g., cephalosporins and carbapenems) and two main classes of new β-lactamase inhibitors: (i) 1,6-diazabicyclo[3,2,1]octane (DBO) analogues (e.g., avibactam and relebactam) and (ii) cyclic boronates (e.g., vaborbactam). Current clinically available options include the carbapenem/β-lactamase inhibitors imipenem/relebactam and meropenem/vaborbactam, which display potent activity against KPC-producing strains, and ceftazidime/avibactam, with this latter combination also covering OXA-48-producers (5). While these additions represent an advance regarding the management of CPE infections, the problem is still far from being resolved, as none of the combinations provide coverage against the growing number of MBL-producing strains, due to a lack of inhibitory potential of those inhibitors against MBLs. Moreover, the increased use of ceftazidime/avibactam worldwide has had an important impact on the evolution of KPC β-lactamases, which can become refractory to the combination through acquisition of amino acid replacements in catalytically important regions, the so-called KPC resistant variants (6).

Other recently approved or late-stage combinations which may mitigate these therapeutic gaps to some extent include aztreonam/avibactam and cefepime/taniborbactam. Aztreonam/avibactam takes advantage of the inefficiency of MBLs to inactivate aztreonam and the high potency of avibactam against serine-type enzymes, thus covering all types of CPE (7). On the other hand, the appearance in the clinical setting of cefepime/taniborbactam, which has already been demonstrated (in the recently published CERTAIN trial) to be superior to meropenem, corresponds to the first combination including a β-lactamase inhibitor with ultrabroad spectrum of β-lactamase coverage, including MBLs (8). However, CPE represent a continuously moving target, and strains showing emerging resistance mechanisms to these combinations are already expanding. Most of these strains include multidrug-resistant *Escherichia coli* lineages (e.g., ST410 and ST167) which are often producing NDM enzymes but also exhibit insertions into their specific penicillin-binding protein 3 (PBP3) (9). These strains are commonly resistant to aztreonam/avibactam but also to all other clinically available or investigational β-lactam/β-lactamase inhibitors, including cefepime/taniborbactam. Thus, new agents active against these emerging threats are urgently needed (10).

A novel ultrabroad-spectrum bicyclic boronate type inhibitor was recently discovered by rational modification of a benzoxaborinine scaffold: xeruborbactam (formerly QPX7728) (11). Xeruborbactam demonstrates a potent and ultrabroad spectrum of β-lactamase coverage, being able to inhibit class A, B, C, and D enzymes in the nanomolar range. Xeruborbactam also outperforms other inhibitors in acting against key enzymatic targets at the biochemical level, such as vaborbactam against KPC enzymes and avibactam against OXA-48-like variants (12). Moreover, xeruborbactam has proven potent biochemical activity and *in vitro* potency against MBLs, including IMP-like enzymes and NDM-9 variants, which are resistant to the inhibitory action of taniborbactam (13–15). Xeruborbactam is currently undergoing evaluation in clinical trials with different intravenous and orally available β-lactams. Its potency in previous microbiological studies has mainly been in combination with meropenem (16). Most of these studies provide data obtained on large collections of randomly selected clinical strains isolated in large surveillance studies (17). Thus, in order to obtain more information about its therapeutic potential against CPE and aid in its precise therapeutic positioning, we evaluated the activity of xeruborbactam against a large collection of isogenic *E. coli* strains with defined β-lactamase content, as well as against large collections of genetically unrelated and whole-genome sequencing (WGS)-characterized CPE strains, including collections of isolates being resistant to combinations such as ceftazidime/avibactam, aztreonam/avibactam, and cefepime/taniborbactam.

## RESULTS AND DISCUSSION

### Activity of meropenem/xeruborbactam against *E. coli* isogenic strains

We first evaluated the antimicrobial activity of meropenem/xeruborbactam and comparator agents against an isogenic panel of *E. coli* strains producing single or double β-lactamases under wild-type and low-permeability conditions (Table 1). Meropenem/xeruborbactam demonstrated near-total activity (MIC range: ≤0.06–16 mg/L; 92/94 susceptible strains) against the collection of *E. coli* laboratory transformants, although some notable effects on its activity were noted in relation to the class of β-lactamase produced and the permeability status. This high potency is likely explained by the strong activity of xeruborbactam against all β-lactamases but could also be due to its previously observed intrinsic antibacterial activity against *E. coli*, where xeruborbactam alone has demonstrated MICs ranging from 2 to 32 mg/L (18).

**TABLE 1:** Antibiotic susceptibility data for newly developed β-lactam/β-lactamase inhibitor combinations against isogenic *E. coli* TG1 and HB4 transformants expressing the most relevant class A, B, C, and D β-lactamases and double carbapenemases found in Enterobacterales under wild-type and low-permeability conditions

Strain	Ambler class	Main phenotype	MIC (mg/L)^*a*^^,^^*b*^^,^^*c*^																
			*E. coli* TG1									*E. coli* HB4							
			MEM (R > 8)	M/X (R > 8)	M/V (R > 8)	I/R (R > 2)	F/T (R > 4)	CAZ (R > 4)	C/A (R > 8)	A/A (R > 4)		MEM (R > 8)	M/X (R > 8)	M/V (R > 8)	I/R (R > 2)	F/T (R > 4)	CAZ (R > 4)	C/A (R > 8)	A/A (R > 4)
*E. coli* TG1	–	Wild-type	≤0.06	≤0.06	≤0.06	0.125	≤0.06	0.125	0.125	≤0.06		–	–	–	–	–	–	–	–
*E. coli* HB4	–	OmpF- and OmpC-deficient	–	–	–	–	–	–	–	–		≤0.06	≤0.06	≤0.06	0.125	0.5	1	0.5	0.125
GES-1	A	ESBL	≤0.06	≤0.06	≤0.06	0.125	≤0.06	4	0.125	≤0.06		≤0.06	≤0.06	≤0.06	0.125	0.5	16	1	0.125
GES-5	A	Carbapenemase	≤0.06	≤0.06	≤0.06	0.125	≤0.06	0.25	0.125	≤0.06		2	≤0.06	≤0.06	0.125	0.5	2	0.5	0.125
GES-7	A	ESBL	≤0.06	≤0.06	≤0.06	0.25	≤0.06	32	0.25	≤0.06		≤0.06	≤0.06	≤0.06	0.125	0.5	>64	1	0.125
GES-20	A	Carbapenemase	≤0.06	≤0.06	≤0.06	0.25	≤0.06	0.25	0.125	≤0.06		2	≤0.06	≤0.06	0.125	0.5	2	0.5	0.125
CTX-M-9	A	ESBL	≤0.06	≤0.06	≤0.06	0.125	≤0.06	0.5	0.125	≤0.06		0.25	≤0.06	≤0.06	0.125	0.5	4	0.5	0.125
CTX-M-15	A	ESBL	≤0.06	≤0.06	≤0.06	0.125	≤0.06	32	0.125	≤0.06		0.25	≤0.06	≤0.06	0.125	1	>64	1	0.125
SHV-12	A	ESBL	≤0.06	≤0.06	≤0.06	0.125	≤0.06	>64	0.25	0.125		0.25	≤0.06	≤0.06	0.125	1	>64	4	0.5
PER-1	A	ESBL	≤0.06	≤0.06	≤0.06	0.125	≤0.06	>64	2	2		≤0.06	≤0.06	≤0.06	≤0.06	1	>64	16	16
VEB-1	A	ESBL	≤0.06	≤0.06	≤0.06	0.125	≤0.06	>64	0.25	≤0.06		≤0.06	≤0.06	≤0.06	≤0.06	0.5	>64	2	1
VEB-25	A	ESBL	≤0.06	≤0.06	≤0.06	≤0.06	≤0.06	>64	64	16		0.125	≤0.06	≤0.06	≤0.06	1	>64	>64	64
BEL-1	A	ESBL	≤0.06	≤0.06	≤0.06	0.125	≤0.06	4	0.125	≤0.06		0.125	≤0.06	≤0.06	≤0.06	1	32	0.5	≤0.06
SFO-1	A	ESBL	≤0.06	≤0.06	≤0.06	≤0.06	0.125	16	0.125	≤0.06		0.25	≤0.06	≤0.06	≤0.06	0.5	32	1	≤0.06
KPC-2	A	Carbapenemase	1	≤0.06	≤0.06	0.25	≤0.06	16	0.25	0.125		>64	≤0.06	0.125	0.5	1	>64	2	1
KPC-35	A	ESBL	≤0.06	≤0.06	≤0.06	0.125	≤0.06	>64	8	≤0.06		0.5	≤0.06	0.25	0.125	2	>64	32	0.25
KPC-3	A	Carbapenemase	1	≤0.06	≤0.06	0.125	≤0.06	32	0.25	≤0.06		32	≤0.06	≤0.06	0.125	1	>64	4	0.125
KPC-31	A	ESBL	≤0.06	≤0.06	≤0.06	0.125	≤0.06	64	16	≤0.06		0.25	≤0.06	0.125	0.125	2	>64	64	0.25
VIM-1	B	Carbapenemase	0.5	≤0.06	0.5	2	1	>64	>64	≤0.06		16	≤0.06	16	2	8	>64	>64	0.125
VIM-2	B	Carbapenemase	≤0.06	≤0.06	≤0.06	0.25	≤0.06	1	1	≤0.06		0.5	≤0.06	0.5	0.25	0.5	8	8	0.125
VIM-4	B	Carbapenemase	0.125	≤0.06	0.125	0.125	≤0.06	8	2	≤0.06		4	≤0.06	4	0.25	0.5	32	8	≤0.06
VIM-20	B	Carbapenemase	≤0.06	≤0.06	≤0.06	0.25	≤0.06	>64	2	≤0.06		2	≤0.06	2	1	0.5	16	16	0.125
IMP-8	B	Carbapenemase	2	≤0.06	0.5	2	32	>64	>64	≤0.06		16	0.25	8	8	>64	>64	>64	≤0.06
IMP-13	B	Carbapenemase	≤0.06	≤0.06	≤0.06	0.25	8	>64	>64	≤0.06		2	≤0.06	2	1	32	>64	>64	0.125
IMP-23	B	Carbapenemase	0.5	0.5	0.5	0.125	8	>64	4	≤0.06		64	16	64	2	>64	>64	>64	≤0.06
IMP-94	B	Carbapenemase	≤0.06	≤0.06	≤0.06	0.25	4	>64	>64	≤0.06		1	≤0.06	1	1	32	>64	>64	0.125
NDM-1	B	Carbapenemase	2	≤0.06	2	1	1	>64	>64	≤0.06		64	4	64	16	16	>64	>64	0.25
NDM-5	B	Carbapenemase	32	≤0.06	32	32	8	>64	>64	≤0.06		>64	1	>64	>64	>64	>64	>64	0.25
NDM-7	B	Carbapenemase	16	≤0.06	16	16	4	>64	>64	≤0.06		>64	2	>64	>64	>64	>64	>64	0.25
NDM-23	B	Carbapenemase	2	≤0.06	2	2	1	>64	>64	≤0.06		32	0.25	32	8	32	>64	>64	0.25
SPM-1	B	Carbapenemase	0.5	0.125	0.25	0.125	0.125	>64	64	≤0.06		32	16	32	0.25	2	>64	>64	≤0.06
AmpC (*K. aerogenes*)	C	AmpC	≤0.06	≤0.06	≤0.06	≤0.06	≤0.06	64	0.25	0.125		4	≤0.06	1	≤0.06	1	>64	2	0.5
AmpC (*E. cloacae*)	C	AmpC	≤0.06	≤0.06	≤0.06	0.125	≤0.06	32	0.125	0.125		0.5	≤0.06	≤0.06	≤0.06	0.5	64	0.5	0.25
CMH-7	C	AmpC	≤0.06	≤0.06	≤0.06	0.125	≤0.06	32	0.125	≤0.06		0.25	≤0.06	≤0.06	0.125	0.5	64	0.5	0.25
CMY-2	C	AmpC	≤0.06	≤0.06	≤0.06	0.25	≤0.06	>64	0.5	0.25		0.5	≤0.06	≤0.06	0.25	0.5	>64	2	1
DHA-1	C	AmpC	≤0.06	≤0.06	≤0.06	0.125	≤0.06	32	0.125	≤0.06		0.25	≤0.06	≤0.06	0.125	0.5	>64	1	0.25
FOX-4	C	AmpC	≤0.06	≤0.06	≤0.06	0.25	≤0.06	>64	1	≤0.06		0.25	≤0.06	≤0.06	0.125	0.5	>64	4	0.25
OXA-1	D	Narrow-spectrum oxacillinase	≤0.06	≤0.06	≤0.06	≤0.06	0.5	0.125	0.125	0.125		0.125	≤0.06	0.125	≤0.06	32	1	0.5	≤0.06
OXA-2	D	Narrow-spectrum oxacillinase	≤0.06	≤0.06	≤0.06	0.125	≤0.06	2	≤0.06	≤0.06		1	≤0.06	1	0.5	0.5	16	1	0.125
OXA-10	D	Narrow-spectrum oxacillinase	≤0.06	≤0.06	≤0.06	0.125	≤0.06	0.125	≤0.06	0.125		2	≤0.06	2	0.25	0.5	2	0.5	1
OXA-14	D	ESBL	≤0.06	≤0.06	≤0.06	0.125	≤0.06	32	4	0.125		0.5	≤0.06	0.5	0.125	1	>64	64	1
OXA-15	D	ESBL	≤0.06	≤0.06	≤0.06	0.125	≤0.06	32	2	≤0.06		0.125	≤0.06	0.125	0.125	1	64	16	0.125
OXA-48	D	Carbapenemase	0.5	≤0.06	0.25	0.25	≤0.06	0.25	≤0.06	≤0.06		8	≤0.06	8	1	0.5	1	0.5	0.25
KPC-3 + VIM-1	A + B	Double carbapenemase	1	≤0.06	0.125	0.5	0.25	64	32	≤0.06		32	≤0.06	8	1	8	>64	>64	0.25
KPC-3 + IMP-94	A + B	Double carbapenemase	1	≤0.06	0.125	0.5	2	64	64	≤0.06		32	≤0.06	8	0.5	16	>64	>64	0.25
KPC-3 + OXA-48	A + D	Double carbapenemase	1	≤0.06	0.125	0.25	≤0.25	16	≤0.25	≤0.06		16	≤0.06	2	0.5	32	>64	0.5	0.25
NDM-1 + OXA-48	B + D	Double carbapenemase	4	≤0.06	4	8	8	>64	>64	≤0.06		>64	2	64	4	32	>64	>64	0.25
OXA-48 + VIM-1	B + D	Double carbapenemase	0.5	≤0.06	0.25	0.5	0.25	>64	32	≤0.06		8	≤0.06	8	2	4	>64	>64	0.25
OXA-48 + IMP-94	B + D	Double carbapenemase	0.25	≤0.06	0.25	1	4	64	32	≤0.06		16	≤0.06	16	1	32	>64	>64	0.25

^*a*^ MEM: meropenem; M/X: meropenem/xeruborbactam; M/V: meropenem/vaborbactam; I/R: imipenem/relebactam; F/T: cefepime/taniborbactam; CAZ: ceftazidime; C/A: ceftazidime/avibactam; A/A: aztreonam/avibactam.

^*b*^ EUCAST breakpoints indicated for Enterobacterales. Clinical breakpoints for combinations that have not yet been approved (meropenem/xeruborbactam and cefepime/taniborbactam) were interpreted using those of the β-lactam partner. Avibactam, relebactam, and taniborbactam were tested at a fixed concentration of 4 mg/L, while vaborbactam and xeruborbactam were tested at 8 mg/L.

^*c*^ –, not applicable.

The *E. coli* TG1 transformants generally yielded meropenem/xeruborbactam MIC values ≤0.06 mg/L. Notably, this high activity was maintained against almost all MBL-producing strains, including those carrying NDMs, against which the addition of xeruborbactam potentiated the activity of meropenem at least 512-fold (from 32 to ≤0.06 mg/L). However, the highest MIC values were observed for the transformants producing SPM-1 (MIC = 0.125 mg/L) and IMP-23 (MIC = 0.5 mg/L). The SPM-1 MBL has been shown to resist the inhibitory activity of xeruborbactam in a recent study, along with other relatively rare MBLs such as SIM-1, PFM-1, and AIM-1. On the other hand, the IMP-23 enzyme also exhibits resistance to xeruborbactam. Interestingly, this variant contains a V67F amino acid substitution that affects a β-hairpin loop in the enzyme’s active site, a modification recently linked to xeruborbactam resistance by Le Terrier et al. (13), following genetic and biochemical analysis of IMP-10, a V67F variant of IMP-1. The IMP-23 enzyme is encoded in the genomes of *Citrobacter freundii* (GenBank accession number NG_049187.1) and *Klebsiella pneumoniae* (GenBank accession number CP097249.1) isolates from China and Taiwan, respectively, whereas the IMP-10 was found to be common in Asia (19). Altogether, these data highlight the need for close monitoring of xeruborbactam-resistant IMP variants and further mechanistic research with these enzymes to gain insights into the evolutionary potential of MBLs toward xeruborbactam resistance. Regarding the rest of the combinations, with the exception of aztreonam/avibactam, the MBL-producing strains (particularly those carrying NDM enzymes) yielded high MIC values for most of the other comparator combinations analyzed, including to some extent the novel combination with anti-MBL activity, cefepime/taniborbactam, as previously observed with recombinant *E. coli* isolates (20). In contrast, aztreonam/avibactam MICs were slightly elevated against transformants producing PER-1 and VEB-25 (MIC = 2 and 16 mg/L, respectively) and to a lesser extent CMY-2 (MIC = 0.25 mg/L), as previously described (21).

Analysis of β-lactamase production in the porin-deficient *E. coli* HB4 host revealed the significant impact of low permeability on the interplay with these enzymes, as demonstrated by the large increase in MICs for most of the combinations tested. This effect was particularly noteworthy for meropenem and enabled a precise analysis of the inhibitory effect of xeruborbactam. Interestingly, xeruborbactam completely restored meropenem activity against most *E. coli* HB4 derivatives, reducing the MIC of meropenem to ≤0.06 mg/L, except for those transformants producing NDM enzymes (for which the MIC ranged from 0.25 to 4 mg/L), and the above-mentioned xeruborbactam-resistant variants SPM-1 (MIC = 16 mg/L) and IMP-23 (MIC = 16 mg/L). The reduced activity of meropenem/xeruborbactam against NDM-producing Enterobacterales with disrupted porins was noted in a previous study (22). The association between porin lesions and production of NDM enzymes also yielded very high MIC values for the cefepime/taniborbactam combination, a phenomenon previously observed in clinical isolates of NDM-producing *K. pneumoniae*, raising further concern about the spectrum of action of these enzymes, particularly when combined with additional mutational resistance mechanisms (5).

### Activity of meropenem/xeruborbactam against clinical CPE strains

**Fig 1:**
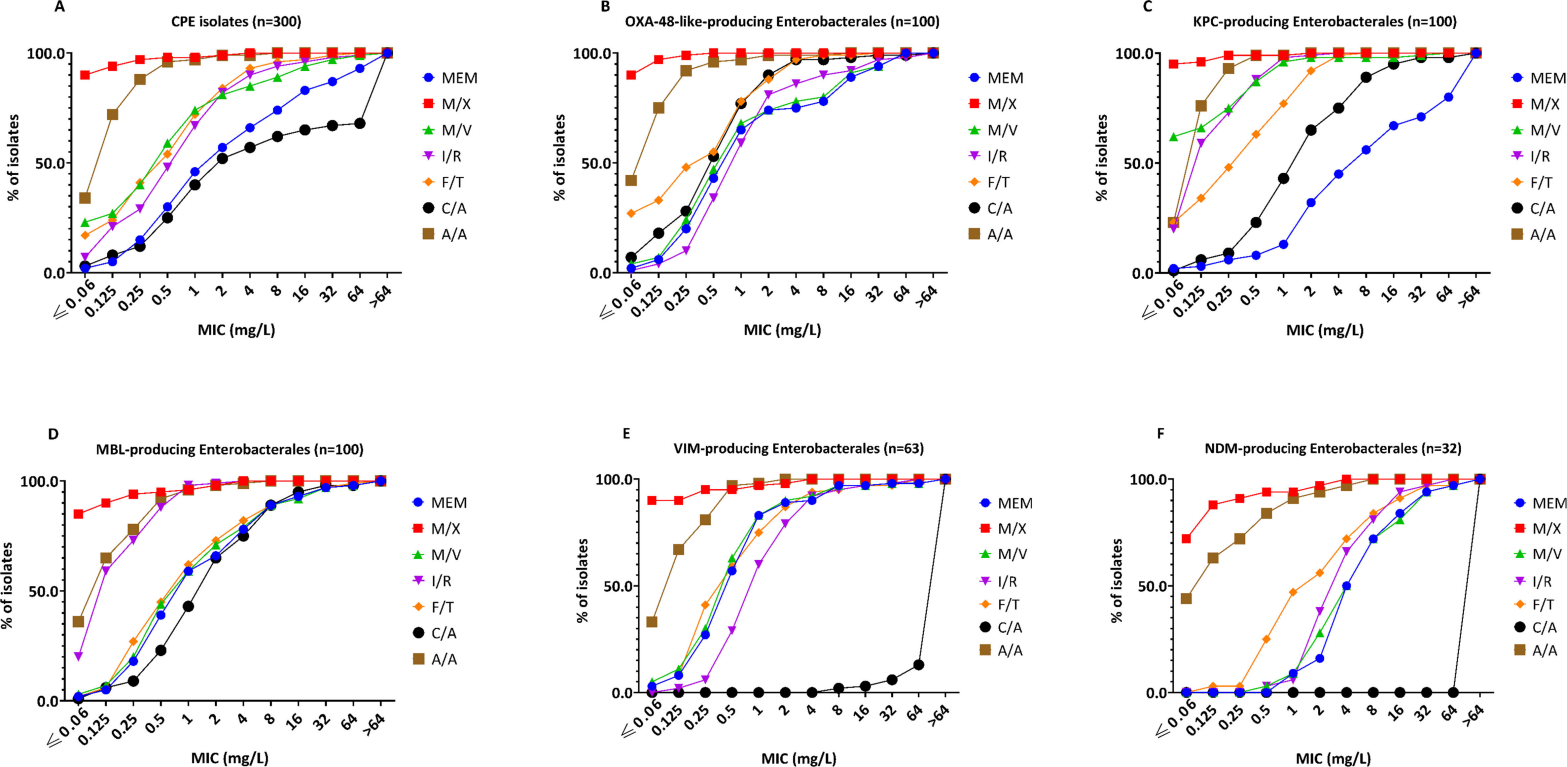
(**A**) Cumulative MIC values of all CPE isolates, (**B**) OXA-48-like-producing isolates, (**C**) KPC-producing isolates, (**D**) MBL-producing isolates. MBL-producing isolates are also divided into (**E**) VIM-producing isolates and (**F**) NDM-producing isolates. MEM: meropenem; M/X: meropenem/xeruborbactam; M/V: meropenem/vaborbactam; I/R: imipenem/relebactam; F/T: cefepime/taniborbactam; C/A: ceftazidime/avibactam; A/A: aztreonam/avibactam.

Detailed MIC values for all 300 clinical strains are shown in Tables S3 to S5. Among the 300 CPE isolates tested, the meropenem/xeruborbactam combination demonstrated exceptional activity, with MIC_50_ and MIC_90_ values ≤ 0.06 mg/L. All isolates were inhibited by meropenem/xeruborbactam at ≤4 mg/L, resulting in 100% susceptibility across the entire collection considering the breakpoint of meropenem alone (Table 2; Fig. 1). The addition of xeruborbactam reduced the meropenem MIC_90_ by more than 1,064-fold (from 64 to ≤0.06 mg/L). When comparing the activity of meropenem/xeruborbactam with that of other combinations active against both serine- and metallo-β-lactamases producers, such as aztreonam/avibactam and cefepime/taniborbactam, we found that the latter combinations retained activity against 99.3% and 92.7% of the collection, respectively, while the others did not exceed 90% activity in any case. This global overview emphasizes the robust potency of meropenem/xeruborbactam, which would effectively cover virtually all CPE currently circulating in Spain.

**TABLE 2:** *In vitro* antibiotic susceptibility activity data for meropenem/xeruborbactam and comparator agents against clinical CPE isolates with defined carbapenemase content^*c*^

Carbapenemase type	Antimicrobial agent(s)^*a*^	MIC (mg/L)												MIC_50_	MIC_90_	Range	% Sus^*b*^	% Res^*b*^
		≤0.06	0.125	0.25	0.5	1	2	4	8	16	32	64	>64					
All (*n* = 300)	MEM	6 (2.0%)	14 (4.7%)	44 (14.7%)	90 (30.0%)	137 (45.7%)	172 (57.3%)	198 (66.0%)	222 (74.0%)	248 (82.7%)	261 (87.0%)	278 (92.7%)	300 (100.0%)	2	64	≤0.06–>64	74.0	26.0
	M/X	270 (90.0%)	283 (94.3%)	292 (97.3%)	294 (98.0%)	295 (98.3%)	298 (99.3%)	300 (100.0%)						≤0.06	≤0.06	≤0.06–4	100.0	0.0
	M/V	68 (22.7%)	80 (26.7%)	119 (39.7%)	178 (59.3%)	223 (74.3%)	243 (81.0%)	255 (85.0%)	267 (89.0%)	281 (93.7%)	290 (96.7%)	298 (99.3%)	300 (100.0%)	0.5	16	≤0.06–>64	89.0	11.0
	I/R	21 (7.0%)	63 (21.0%)	86 (28.7%)	143 (47.7%)	201 (67.0%)	247 (82.3%)	270 (90.0%)	281 (93.7%)	288 (96.0%)	295 (98.3%)	298 (99.3%)	300 (100.0%)	1	4	≤0.06–>64	82.3	17.7
	F/T	52 (17.3%)	73 (24.3%)	122 (40.7%)	163 (54.3%)	217 (72.3%)	253 (84.3%)	278 (92.7%)	288 (96.0%)	292 (97.3%)	292 (97.3%)	299 (99.7%)	300 (100.0%)	0.5	4	≤0.06–>64	92.7	7.3
	C/A	8 (2.7%)	24 (8.0%)	37 (12.3%)	77 (25.7%)	121 (40.3%)	155 (51.7%)	172 (57.3%)	187 (62.3%)	195 (65.0%)	201 (67.0%)	205 (68.3%)	300 (100.0%)	2	>64	≤0.06–>64	62.3	37.7
	A/A	101 (33.7%)	215 (71.7%)	263 (87.7%)	287 (95.7%)	292 (97.3%)	297 (99.0%)	298 (99.3%)	299 (99.7%)	300 (100.0%)				0.125	0.5	≤0.06–16	99.3	0.7
OXA-48-like (*n* = 100)	MEM	2 (2.0%)	6 (6.0%)	20 (20.0%)	43 (43.0%)	65 (65.0%)	74 (74.0%)	75 (75.0%)	78 (78.0%)	89 (89.0%)	94 (94.0%)	100 (100.0%)		1	32	≤0.06–64	78.0	22.0
	M/X	90 (90.0%)	97 (97.0%)	99 (99.0%)	100 (100.0%)									≤0.06	≤0.06	≤0.06–0.5	100.0	0.0
	M/V	4 (4.0%)	7 (7.0%)	24 (24.0%)	47 (47.0%)	68 (68.0%)	74 (74.0%)	78 (78.0%)	80 (80.0%)	91 (91.0%)	94 (94.0%)	100 (100.0%)		1	16	≤0.06–64	80.0	20.0
	I/R	1 (1.0%)	4 (4.0%)	10 (10.0%)	34 (34.0%)	59 (59.0%)	81 (81.0%)	86 (86.0%)	90 (90.0%)	92 (92.0%)	97 (97.0%)	98 (98.0%)	100 (100.0%)	1	8	≤0.06–>64	81.0	19.0
	F/T	27 (27.0%)	33 (33.0%)	48 (48.0%)	55 (55.0%)	78 (78.0%)	88 (88.0%)	97 (97.0%)	99 (99.0%)	99 (99.0%)	100 (100.0%)			0.5	4	≤0.06–32	97.0	3.0
	C/A	7 (7.0%)	18 (18.0%)	28 (28.0%)	53 (53.0%)	77 (77.0%)	90 (90.0%)	97 (97.0%)	97 (97.0%)	98 (98.0%)	99 (99.0%)	99 (99.0%)	100 (100.0%)	0.5	2	≤0.06–>64	97.0	3.0
	A/A	42 (42.0%)	75 (75.0%)	92 (92.0%)	96 (96.0%)	97 (97.0%)	99 (99.0%)	99 (99.0%)	99 (99.0%)	100 (100.0%)				0.125	0.25	≤0.06–16	99.0	1.0
KPC-like (*n* = 100)	MEM	2 (2.0%)	3 (3.0%)	6 (6.0%)	8 (8.0%)	13 (13.0%)	32 (32.0%)	45 (45.0%)	55 (55.0%)	66 (66.0%)	70 (70.0%)	80 (80.0%)	100 (100.0%)	8	>64	≤0.06–>64	55.0	45.0
	M/X	95 (95.0%)	96 (96.0%)	99 (99.0%)	99 (99.0%)	99 (99.0%)	100 (100.0%)							≤0.06	≤0.06	≤0.06–2	100.0	0.0
	M/V	61 (61.0%)	66 (66.0%)	75 (75.0%)	87 (87.0%)	96 (96.0%)	98 (98.0%)	98 (98.0%)	98 (98.0%)	98 (98.0%)	99 (99.0%)	100 (100.0%)		≤0.06	1	≤0.06–64	98.0	2.0
	I/R	20 (20.0%)	58 (58.0%)	72 (72.0%)	88 (88.0%)	98 (98.0%)	99 (99.0%)	100 (100.0%)						0.125	1	≤0.06-4	99.0	1.0
	F/T	23 (23.0%)	34 (34.0%)	47 (47.0%)	63 (63.0%)	77 (77.0%)	92 (92.0%)	99 (99.0%)	100 (100.0%)					0.5	2	≤0.06–8	99.0	1.0
	C/A	1 (1.0%)	6 (6.0%)	9 (9.0%)	24 (24.0%)	44 (44.0%)	65 (65.0%)	75 (75.0%)	89 (89.0%)	95 (95.0%)	98 (98.0%)	98 (98.0%)	100 (100.0%)	2	16	≤0.06–>64	89.0	11.0
	A/A	23 (23.0%)	75 (75.0%)	93 (93.0%)	99 (99.0%)	99 (99.0%)	100 (100.0%)							0.125	0.25	≤0.06–2	100.0	0.0
MBL (*n* = 100)	MEM	2 (2.0%)	5 (5.0%)	18 (18.0%)	39 (39.0%)	59 (59.0%)	66 (66.0%)	78 (78.0%)	89 (89.0%)	93 (93.0%)	97 (97.0%)	98 (98.0%)	100 (100.0%)	1	16	≤0.06–>64	89.0	11.0
	M/X	85 (85.0%)	90 (90.0%)	94 (94.4%)	95 (95.0%)	96 (96.0%)	98 (98.0%)	100 (100.0%)						≤0.06	0.125	≤0.06–4	100.0	0.0
	M/V	3 (3.0%)	7 (7.0%)	20 (20.0%)	44 (44.0%)	59 (59.0%)	71 (71.0%)	79 (79.0%)	89 (89.0%)	92 (92.0%)	97 (97.0%)	98 (98.0%)	100 (100.0%)	1	16	≤0.06–>64	89.0	11.0
	I/R		1 (1.0%)	4 (4.0%)	21 (21.0%)	44 (44.0%)	67 (67.0%)	84 (84.0%)	91 (91.0%)	96 (96.0%)	98 (98.0%)	100 (100.0%)		2	8	0.125–64	67.0	33.0
	F/T	2 (2.0%)	6 (6.0%)	27 (27.0%)	45 (45.0%)	62 (62.0%)	73 (73.0%)	82 (82.0%)	89 (89.0%)	93 (93.0%)	97 (97.0%)	99 (99.0%)	100 (100.0%)	1	16	≤0.06–>64	82.0	18.0
	C/A								1 (1.0%)	2 (2.0%)	4 (4.0%)	8 (8.0%)	100 (100.0%)	>64	>64	8–>64	1.0	99.0
	A/A	36 (36.0%)	65 (65.0%)	78 (78.0%)	92 (92.0%)	96 (96.0%)	98 (98.0%)	99 (99.0%)	100 (100.0%)					0.125	0.5	≤0.06–8	99.0	1.0
VIM (*n* = 63)	MEM	2 (3.2%)	5 (7.9%)	17 (27.0%)	36 (57.1%)	52 (82.5%)	56 (88.9%)	57 (90.5%)	61 (96.8%)	61 (96.8%)	62 (98.4%)	62 (98.4%)	63 (100.0%)	0.5	4	≤0.06–>64	96.8	3.2
	M/X	57 (90.5%)	57 (90.5%)	60 (95.2%)	60 (95.2%)	61 (96.8%)	62 (98.4%)	63 (100.0%)						≤0.06	≤0.06	≤0.06–4	100.0	0.0
	M/V	3 (4.8%)	7 (11.1%)	19 (30.2%)	40 (63.5%)	52 (82.5%)	57 (90.5%)	58 (92.1%)	61 (96.8%)	61 (96.8%)	62 (98.4%)	62 (98.4%)	63 (100.0%)	0.5	2	≤0.06–>64	96.8	3.2
	I/R		1 (1.6%)	4 (6.3%)	18 (28.6%)	38 (60.3%)	50 (79.4%)	58 (92.1%)	60 (95.2%)	61 (96.8%)	62 (98.4%)	63 (100.0%)		1	4	0.125–64	79.4	20.6
	F/T	2 (3.2%)	5 (7.9%)	26 (41.3%)	37 (58.7%)	47 (74.6%)	55 (87.3%)	59 (93.7%)	60 (95.2%)	61 (96.8%)	61 (96.8%)	63 (100.0%)		0.5	4	≤0.06–64	93.7	6.3
	C/A								1 (1.6%)	2 (3.2%)	4 (6.3%)	8 (12.7%)	63 (100.0%)	>64	>64	8–>64	1.6	98.4
	A/A	21 (33.3%)	42 (66.7%)	51 (81.0%)	61 (96.8%)	62 (98.4%)	63 (100.0%)							0.125	0.5	≤0.06–2	100.0	0.0
NDM (*n* = 32)	MEM					3 (9.4%)	5 (15.6%)	16 (50.0%)	23 (71.9%)	27 (84.4%)	30 (93.8%)	31 (96.9%)	32 (100.0%)	4	32	1–>64	71.9	28.1
	M/X	23 (71.9%)	28 (87.5%)	29 (90.6%)	30 (93.8%)	30 (93.8%)	31 (96.9%)	32 (100.0%)						≤0.06	0.25	≤0.06-4	100.0	0.0
	M/V				1 (3.1%)	3 (9.4%)	9 (28.1%)	16 (50.0%)	23 (71.9%)	26 (81.3%)	30 (93.8%)	31 (96.9%)	32 (100.0%)	4	32	0.5–>64	71.9	28.1
	I/R				1 (3.1%)	2 (6.3%)	12 (37.5%)	21 (65.6%)	26 (81.3%)	30 (93.8%)	31 (96.9%)	32 (100.0%)		4	16	0.5–64	37.5	62.5
	F/T		1 (3.1%)	1 (3.1%)	8 (25.0%)	15 (46.9%)	18 (56.3%)	23 (71.9%)	27 (84.4%)	29 (90.6%)	31 (96.9%)	31 (96.9%)	32 (100.0%)	2	16	0.125–>64	71.9	28.1
	C/A												32 (100.0%)	>64	>64	>64–>64	0.0	100.0
	A/A	14 (43.8%)	20 (62.5%)	23 (71.9%)	27 (84.4%)	29 (90.6%)	30 (93.8%)	31 (96.9%)	32 (100.0%)					0.125	1	≤0.06–8	96.9	3.1

^*a*^ MEM: meropenem; M/X: meropenem/xeruborbactam; M/V: meropenem/vaborbactam; I/R: imipenem/relebactam; F/T: cefepime/taniborbactam; C/A: ceftazidime/avibactam; A/A: aztreonam/avibactam.

^*b*^ EUCAST breakpoints indicated for Enterobacterales. Clinical breakpoints for combinations that have not yet been approved (meropenem/xeruborbactam and cefepime/taniborbactam) were interpreted using those of the β-lactam partner.

^*c*^ Empty cells indicate the cumulative percentage of isolates already reaches 100% at lower MIC values for each combination.

Meropenem/xeruborbactam was the most active combination against OXA-48-like-producing Enterobacterales. For this subset of strains, the MIC_50_ and MIC_90_ values were ≤0.06 mg/L, with the MIC range significantly reduced compared to the whole collection. All strains within this subset were inhibited at ≤0.5 mg/L. The enhanced activity can be attributed to three factors: (i) the already relatively high activity of meropenem against OXA-48-producing strains, facilitating the achievement of lower MIC values upon addition of the inhibitor (23); (ii) the strong inhibitory activity of xeruborbactam against the OXA-48 enzyme, which is inhibited at very low *K_i_*_app_ values (in the nanomolar range) (24); and (iii) the antibacterial activity of xeruborbactam in Enterobacterales (18). Aztreonam/avibactam, ceftazidime/avibactam, and cefepime/taniborbactam also showed high activity against this subset of strains. As expected, imipenem/relebactam and meropenem/vaborbactam were the least effective combinations, as both relebactam and vaborbactam show very poor inhibitory activity against the OXA-48 enzyme (both combinations showed susceptibility rates around 80% against the whole subset of clinical OXA-48-producing Enterobacterales) (25).

KPC-producing Enterobacterales represent a therapeutic target against which the greatest number of treatment options are available, as all inhibitors included in commercially available or investigational β-lactam/β-lactamase inhibitor combinations tested here effectively block this serine carbapenemase (26). In line with these findings, aztreonam/avibactam, meropenem/vaborbactam, imipenem/relebactam, cefepime/taniborbactam, and, to a lesser extent, ceftazidime/avibactam were highly active, with susceptibility rates ranging from 89% to 100%. However, meropenem/xeruborbactam once again proved to be the most active combination, demonstrating 100% susceptibility across all isolates. Interestingly, meropenem/xeruborbactam showed superior activity to carbapenem/β-lactamase inhibitor combinations such as imipenem/relebactam and meropenem/vaborbactam, which have been highlighted in recently available Infectious Diseases Society of America (IDSA) guidelines as promising options for combating high-inoculum KPC-producing Enterobacterales infections, such as ventilator-associated pneumonia, related to an increased risk of therapeutic failure and resistance development with ceftazidime/avibactam (27).

Meropenem/xeruborbactam showed the greatest promise in the challenging therapeutic niche of MBL-producing Enterobacterales, with MIC_50_ and MIC_90_ values of ≤0.06 and 0.125 mg/L respectively, 100% susceptibility rates, and MICs ranging from ≤0.06 to 4 mg/L. The high activity was maintained irrespective of the specific MBL produced, although the potency was lower against MBL producers than against KPC or OXA-48 producers. Notably, meropenem/xeruborbactam has been shown to retain almost 100% activity against large collections of MBL-producing Enterobacterales strains in global surveys conducted between 2014 and 2017 (17). The present findings further support and extend these previous findings, demonstrating the continued effectiveness of meropenem/xeruborbactam against strains circulating between 2017 and 2024, including genetically diverse MBL-producing Enterobacterales*.* As observed in previous studies, some strains yielded MICs in the top range of susceptibility (4 mg/L). These elevated MICs have been previously associated with the production of MBL enzymes and disrupted porin channels, which together have been shown to reduce the potency of the combination by eight to 16 times (22). This observation is further corroborated by our *E. coli* HB4 experimental model, in which NDM-producing transformants with porin deficiency exhibited MICs of 4 mg/L. Aztreonam/avibactam and, to a lesser extent, cefepime/taniborbactam also displayed high activity against the MBL-producing strains included in this study. However, two strains showed decreased susceptibility or resistance to aztreonam/avibactam (MICs = 4-8 mg/L), and 18 strains were resistant to cefepime/taniborbactam (MICs = 8-64 mg/L), further emphasizing the need for developing xeruborbactam-based combinations to expand treatment options against MBL producers.

### Activity against clinical strains resistant to ceftazidime/avibactam, aztreonam/avibactam, and/or cefepime/taniborbactam

The genotypic features and activity of meropenem/xeruborbactam against a panel of 14 clinical isolates carrying KPC amino acid variants are shown in Table 3. Most of these strains were recovered from patients who had previously received courses of treatment with ceftazidime/avibactam (data not shown), and in most cases, they belong to the worldwide scattered ST512 *K. pneumoniae* lineage, which became disseminated throughout Spain after its introduction from Italy in a large-scale outbreak that occurred in Southern Spain in 2012 (28). The panel was enriched with unduplicated variants in which the ceftazidime/avibactam resistance phenotype was driven by different alterations (substitutions, deletions, or large insertions) in different catalytic hot spots of the KPC architecture, including the classic Ω-loop, but also other distant regions, such as the 240-loop or the 270-loop (29). These strains were in all cases resistant to ceftazidime/avibactam (14/14; MICs = 16–>64 mg/L) and in some cases to cefepime/taniborbactam (5/14; MICs = 0.125–16 mg/L) or aztreonam/avibactam (1/14; MICs = 0.125–64 mg/L). Moreover, while ceftazidime/avibactam-resistant KPC variants are commonly associated with decreased activity against carbapenems, half of the strains producing these variants exhibited meropenem resistance (7/14) due to a disruption of OmpK35 and the classic GD insertion in OmpK36, a genetic hallmark of the ST512 lineage (6). Interestingly, the novel combinations meropenem/vaborbactam and imipenem/relebactam exhibited 100% susceptibility rates and high potency against all strains, particularly imipenem/relebactam, with MICs ranging from ≤0.06 to 2 mg/L and from ≤0.06 to 0.5 mg/L, respectively. However, meropenem/xeruborbactam outperformed these two combinations, with most strains yielding MICs of ≤0.06 mg/L (10/14) and in no case exceeding 0.25 mg/L. It is tempting to suggest that the increased potency of the meropenem/xeruborbactam combination against these strains may result from the following possible scenarios: (i) the combination takes advantage of including a carbapenem partner, against which these variants usually exhibit decreased hydrolytic activity (30), and (ii) xeruborbactam inhibits wild-type KPC enzymes at lower concentrations than vaborbactam and relebactam, and thus, the findings suggest that the increased potency is maintained against these variants (12). Altogether, these findings highlight meropenem/xeruborbactam as an attractive combination to combat strains showing KPC-mediated ceftazidime/avibactam resistance.

**TABLE 3:** Genotypic and phenotypic features of a collection of CPE producing different KPC variants against meropenem/xeruborbactam and comparator agents

Isolate id	Species	MLST	Resistance mechanisms*^a^*						MIC (mg/L)*^b^^,^^c^*						
			KPC β-lactamase	KPC amino acid alteration	Location in sequence*^d^*	OmpK35	OmpK36		MEM (R > 8)	M/X (R > 8)	M/V (R > 8)	I/R (R > 2)	F/T (R > 4)	C/A (R > 8)	A/A (R > 4)
20220166	*K. pneumoniae*	512	KPC-23	V240A	Loop 237-243	C66fs	–		4	≤0.06	≤0.06	≤0.06	0.125	16	0.25
231992	*K. pneumoniae*	512	KPC-28	del_242-243_GT	Loop 237-243	C66fs	ins_114_GD		1	≤0.06	0.5	≤0.06	16	>64	4
20211109	*K. pneumoniae*	512	KPC-31	D179Y	Ω-loop	C66fs	–		2	≤0.06	1	0.125	8	>64	0.5
KPLA	*K. pneumoniae*	258	KPC-35	L169P	Ω-loop	C66fs	ins_114_GD		1	≤0.06	0.5	0.25	4	32	0.25
183111	*K. pneumoniae*	512	KPC-39	A172T	Ω-loop	C66fs	ins_114_GD		16	0.125	2	0.25	4	64	1
183860	*K. pneumoniae*	512	KPC-47	A172T, T243A	Ω-loop and loop 237-243	C66fs	ins_114_GD		32	≤0.06	0.5	0.5	4	>64	0.5
183358	*K. pneumoniae*	512	KPC-48	L169P, A172T	Ω-loop	C66fs	ins_114_GD		8	0.25	1	0.5	8	>64	2
20220294	*K. pneumoniae*	512	KPC-66	del_168-169_EL	Ω-loop	C66fs	ins_114_GD		4	0.25	1	0.25	1	32	0.125
202826	*K. pneumoniae*	512	KPC-85	A172V	Ω-loop	C66fs	ins_114_GD		64	≤0.06	1	0.125	4	32	0.5
171513	*K. pneumoniae*	512	KPC-94	L169H, del_170_N	Ω-loop	C66fs	ins_114_GD		1	≤0.06	1	0.125	2	>64	64
180510	*K. pneumoniae*	512	KPC-95	A172T, D179Y	Ω-loop	C66fs	ins_114_GD		16	0.25	1	0.5	16	>64	4
20220366	*K. pneumoniae*	512	KPC-132	ins_270_KDDKSRAPN	Loop 266-275	C66fs	ins_114_GD		16	≤0.06	2	0.25	4	>64	0.25
196228	*K. pneumoniae*	512	KPC-148	ins_275_EAVYTRAPNKDDKYS	Loop 266-275	C66fs	ins_114_GD		32	≤0.06	1	0.125	8	>64	1
231595	*K. pneumoniae*	512	KPC-178	P174L	Ω-loop	C66fs	ins_114_GD		32	≤0.06	0.5	0.125	4	64	0.5

^*a*^ –, not found; del: deletion of amino acids; fs: frameshift; ins: insertion of amino acids.

^*b*^ MEM: meropenem; M/X: meropenem/xeruborbactam; M/V: meropenem/vaborbactam; I/R: imipenem/relebactam; F/T: cefepime/taniborbactam; C/A: ceftazidime/avibactam; A/A: aztreonam/avibactam.

^*c*^ EUCAST breakpoints indicated for Enterobacterales. Clinical breakpoints for combinations that have not yet been approved (meropenem/xeruborbactam and cefepime/taniborbactam) were interpreted using those of the β-lactam partner. Avibactam, relebactam, and taniborbactam were tested at a fixed concentration of 4 mg/L, while vaborbactam and xeruborbactam were tested at 8 mg/L.

^*d*^ Location in KPC loops previously indicated by Hobson C. A. et al. AAC 2022 (31).

Finally, a comprehensive analysis of the activity of meropenem/xeruborbactam against strains showing resistance to newly developed β-lactam/β-lactamase inhibitor combinations, along with their most relevant genotypic features, is summarized in Table 4. These strains comprised 18 Enterobacterales isolates (4 *K. pneumoniae*, 13 *E. coli*, and 1 *Enterobacter cloacae* complex) carrying different combinations of β-lactamases (including mostly MBLs or recently described inhibitor-resistant extended-spectrum β-lactamases (ESBLs), such as VEB-25) (32) in most cases combined with additional chromosomal mutations affecting porin channels, efflux pump activity, and, more importantly, the β-lactam target protein PBP3. The strains were commonly resistant to meropenem, meropenem/vaborbactam, imipenem/relebactam, and ceftazidime/avibactam, as they lack activity against MBL producers. More concerning, they yielded very high MICs for aztreonam/avibactam (15/18 resistant; MICs = 1–>64 mg/L) and cefepime/taniborbactam (14/18 resistant; MIC range = 1–>64 mg/L). Meropenem/xeruborbactam demonstrated 100% activity against this challenging panel, reducing the MIC of meropenem to values between ≤0.06 and 0.25 mg/L except for one *K. pneumoniae* isolate, which lacked carbapenemases but exhibited a disrupted OmpK36 (MIC = 4 mg/L). Probably, the most remarkable finding is that xeruborbactam restored the activity of meropenem against all *E. coli* isolates carrying the YRIN or YRIK tetra amino acid insertions in PBP3. Interestingly, this PBP3 alteration is increasingly described in high-risk carbapenemase-producing *E. coli* lineages and induces changes in the transpeptidase domain of the PBP enzyme. This alteration significantly reduces the activity of β-lactams whose spectrum of PBP blockade is highly restricted to PBP3, such as aztreonam and cefepime, and thus their respective combinations (33, 34). However, xeruborbactam can restore meropenem susceptibility against these strains because it is able to inactivate the underlying carbapenemase and because meropenem and xeruborbactam are able to target multiple PBPs in *E. coli*, thus bypassing the deleterious effect of PBP3 modifications on the antibacterial activity of aztreonam, cefepime, and their respective combinations (18, 35). Altogether, our findings provide an important baseline of *in vitro* data for the positioning of meropenem/xeruborbactam as a promising antibacterial combination for combating CPE, including emerging strains showing resistance to other newly developed β-lactam/β-lactamase inhibitor combinations, and argue for continuing its clinical development.

**TABLE 4:** Genotypic and phenotypic features of a collection of isolates showing emerging resistance mechanisms to newly developed β-lactam/β-lactamase inhibitor combinations against meropenem/xeruborbactam and comparator agents

Isolate ID	Species	MLST	Resistance mechanisms*^a^*							MIC (mg/L) *^b^^,^^c^*						
			Transferable		Chromosomal											
			Carbapenemases	Other β-lactamases	PBP3 insertion	OmpF-like^*d*^	OmpC-like^*d*^	*acrR*	*ramR*	MEM (R > 8)	M/X (R > 8)	M/V (R > 8)	I/R (R > 2)	F/T (R > 4)	C/A (R > 8)	A/A (R > 4)
R236	*K. pneumoniae*	14	VIM-1	SHV-5	–	–	ins_114_GD	–	M101fs	64	2	64	16	64	>64	8
N3418	*K. pneumoniae*	323	KPC-2	OXA-10, SHV-1, VEB-25	–	–	–	–	–	16	≤0.06	≤0.06	0.125	1	64	16
N3644	*K. pneumoniae*	37	–	CMY-4, SHV-11	–	–	W125*	–	–	16	4	8	16	2	>64	>64
N3041	*K. pneumoniae*	1473	–	DHA-1, TEM-1	–	–	–	S215fs	–	4	≤0.06	1	0.125	16	2	>64
R5989	*E. coli*	471	NDM-5, OXA-181	CMY-42, CTX-M-15, TEM-1	YRIN	–	–	–	–	16	0.25	16	4	16	>64	16
R2222	*E. coli*	471	NDM-4	CMY-42, CTX-M-15, OXA-1, TEM-1	YRIK	–	–	–	–	8	≤0.06	8	4	32	>64	16
N1416	*E. coli*	477	NDM-5	CTX-M-15, OXA-1, TEM-1	YRIK	T263fs	–	–	–	16	0.125	16	8	>64	>64	8
N1110	*E. coli*	471	–	CMY-42, CTX-M-15	YRIK	–	–	–	–	≤0.06	≤0.06	≤0.06	0.125	4	4	>64
R460	*E. coli*	648*^e^*	NDM-5	CMY-6, CTX-M-15	YRIN	T263fs	–	–	–	32	0.125	16	8	32	>64	8
N1606	*E. coli*	650	KPC-3, NDM-5	CMY-145, CTX-M-15, OXA-1, SHV-11, TEM-1	YRIN	–	–	–	–	64	0.125	32	4	64	>64	8
R461	*E. coli*	2	NDM-1	CMY-6	YRIN	–	–	V29fs	–	32	0.25	32	8	64	>64	16
R3033	*E. coli*	477	NDM-5	CMY-42-A171S, TEM-1	YRIK	Y253fs	–	–	–	16	≤0.06	16	8	64	>64	16
R5987	*E. coli*	477	NDM-5	CMY-42-A171S, TEM-1	YRIK	Y253fs	–	–	–	16	≤0.06	16	8	32	>64	16
N951	*E. coli*	477	NDM-5	CMY-42-A171S, TEM-1	YRIK	Y253fs	–	–	–	8	≤0.06	8	8	64	>64	16
ARGA00338	*E. coli*	471	OXA-484	CMY-42, TEM-1	YRIN	–	–	–	–	0.125	≤0.06	≤0.06	0.125	1	4	4
AI2843	*E. coli*	410	NDM-5	CMY-2, CTX-M-15, OXA-1, TEM-1	YRIN	–	–	–	–	4	≤0.06	4	8	16	>64	1
AI2858	*E. coli*	648	NDM-7	CMY-6, CTX-M-15, OXA-1	YRIN	–	–	–	–	8	≤0.06	8	8	8	>64	4
N3046	*E. cloacae* complex	50	–	ACT-15	–	–	–	–	–	2	0.125	1	0.5	16	8	8

^*a*^ –, not found; *: stop codon; ins: insertion of amino acids; fs: frameshift; YRIN: four amino acid insertion of tyrosine-arginine-isoleucine-asparagine in the PBP3 codifying-sequence; YRIK: four amino acid insertion of tyrosine-arginine-isoleucine-lysine in the PBP3 codifying-sequence.

^*b*^ MEM: meropenem; M/X: meropenem/xeruborbactam; M/V: meropenem/vaborbactam; I/R: imipenem/relebactam; F/T: cefepime/taniborbactam; C/A: ceftazidime/avibactam; A/A: aztreonam/avibactam.

^*c*^ EUCAST breakpoints indicated for Enterobacterales. Clinical breakpoints for combinations that have not yet been approved (meropenem/xeruborbactam and cefepime/taniborbactam) were interpreted using those of the β-lactam partner. Avibactam, relebactam, and taniborbactam were tested at a fixed concentration of 4 mg/L, while vaborbactam and xeruborbactam were tested at 8 mg/L.

^*d*^ OmpF-like: OmpK35 for *K. pneumoniae*, OmpF for *E. coli* and OmpE35 for *E. cloacae* complex; OmpC-like: OmpK36 for *K. pneumoniae*, OmpC for *E. coli*, and OmpE36 for *E. cloacae* complex.

^*e*^ Could not be determined following Pasteur scheme. Assigned to ST648 following the Achtman scheme.

## MATERIALS AND METHODS

### Recombinant isolates

In order to elucidate the specific impact of acquired β-lactamases circulating in Enterobacterales on meropenem/xeruborbactam and other broad-spectrum comparator β-lactam/β-lactamase inhibitor combinations (meropenem/vaborbactam, imipenem/relebactam, cefepime/taniborbactam, ceftazidime/avibactam, and aztreonam/avibactam) and also to examine the interaction between β-lactamase production and decreased outer membrane permeability on the activity of these combinations, we utilized a previously engineered library of *E. coli* recombinant isolates (36). For this work, the collection was enriched with 22 additional transformants, including producers of specific inhibitor-resistant β-lactamases. To this end, 11 new β-lactamase genes were cloned under conditions of wild-type (*E. coli* TG1) and low (*E. coli* HB4; OmpF- and OmpC-deficient) permeability, resulting in a final collection of 94 laboratory transformants. The genes *bla*_VEB-25_, *bla*_BEL-1_, *bla*_SFO-1_, *bla*_VIM-4_, *bla*_IMP-8_, *bla*_IMP-23_, *bla*_SPM-1_, *bla*_CMH-7_, *bla*_AmpC (*Enterobacter cloacae*)_, *bla*_AmpC (*Klebsiella aerogenes*)_, and *bla*_OXA-1_ were ligated to the pUCP-24 plasmid and electroporated into both *E. coli* hosts. The isolates were selected on Luria-Bertani (LB) agar plates containing 10 mg/L gentamicin. All transformants were confirmed by PCR, plasmid restriction analysis, Sanger sequencing, and MIC determination.

### Clinical isolates

This study included a representative collection of 300 non-duplicated clinical isolates of CPE recovered from patients admitted to 39 Spanish hospitals across 13 different Spanish regions during the period 2017–2024. The strains were collected by researchers from three reference laboratories in Northern, Central, and Southern Spain, each involved in a distinct large-scale surveillance initiative: (i) the Carbapenem-Resistant Enterobacterales, Genome Database Project (Microbiology Department, Complexo Hospitalario Universitario A Coruña, A Coruña) (37); (ii) the Spanish Reference Laboratory for Research in Antibiotic Resistance and Healthcare-associated Infections, Centro Nacional de Microbiología, Madrid (38); and (iii) the Andalusian Reference PIRASOA Surveillance Program for the Prevention and Control of Healthcare-Associated Infections (PIRASOA Reference Laboratory, Hospital Universitario Virgen Macarena, Seville) (39).

From the collections obtained in these three reference laboratories, the final selection of isolates evaluated here included at least 100 representatives of each of the three main types of circulating carbapenemases (OXA-48-like, KPC-like, and MBLs). The 300 isolates were selected by a detailed selection process aimed at maximizing the diversity of bacterial species, multi-locus sequence types, and β-lactamase content, to guarantee the representativeness of the collection. A summary and detailed list of the isolates (including bacterial species, sequence types and β-lactamase content are available at Tables S1 to S5). In these three centers, preliminary characterization of carbapenemase production was carried out using phenotypic methods (such as the modified carbapenem inactivation method [mCIM] and Carba NP tests) and molecular techniques (PCR and Sanger sequencing). The bacterial species, sequence type, and carbapenemase content were further confirmed by WGS using short-read Illumina technology. All clinical strains and their corresponding *FASTQ* files were centralized at a reference center (Microbiology Department, Complexo Hospitalario Universitario A Coruña) for antimicrobial susceptibility testing and detailed WGS resistome analysis (described below).

Additionally, two challenging sets of strains previously collected by researchers and exhibiting emerging resistance mechanisms to β-lactams were included: (i) a collection of 14 *K. pneumoniae* isolates carrying KPC β-lactamase variants associated with high-level ceftazidime/avibactam resistance, most of which were recovered from patients previously treated with this combination, and (ii) a collection of 18 clinical Enterobacterales isolates from different bacterial species, exhibiting various emerging resistance mechanisms (e.g., PBP3 insertions and other chromosomal alterations in combination with MBLs or inhibitor-resistant ESBLs) associated with resistance or decreased susceptibility to new combinations with activity against metallo-β-lactamases, specifically aztreonam/avibactam and/or cefepime/taniborbactam (40).

### Resistance genomics

For strains that had already been sequenced, paired-end Illumina reads were sent to the reference laboratory for analysis, and the other strains were sequenced *ad hoc* on an Illumina platform for this study. Briefly, the quality control of all paired-end Illumina reads was performed using fastp (v.0.32.2) (41) and BMTagger (v.3.1) (42). Genome assembly was carried out with Unicycler (v.0.5.0) (43). To assess quality, completeness, and the absence of contamination, the assembled genomes were analyzed with CheckM (v.1.1.3) (44). Species were identified using Kmerfinder (v.3.2) (45) and multilocus sequence typing (MLST) (Center for Genomic Epidemiology, v.2.0) (46). Putative open reading frames were annotated with Bakta (v.1.7.0) (47), and horizontally acquired resistance determinants were characterized using Resistance Gene Identifier (RGI) (v.5.2.0) and Comprehensive Antibiotic Resistance Database (CARD) (v.3.2.8) (48).

For isolates producing KPC β-lactamase variants, amino acid sequence variation in the KPC enzymes was manually analyzed using BLAST (49). KPC amino acid numbering followed the Ambler scheme. For isolates showing emerging resistance mechanisms to aztreonam/avibactam and cefepime/taniborbactam, the presence of chromosomal mutations in genes encoding PBPs, porins, efflux pump regulators, and iron uptake-related genes was further analyzed by variant calling with Snippy (v.4.6.0) (50), and using the genome of the β-lactam susceptible strains *K. pneumoniae* ATCC 10031, *E. coli* ATCC 25922 and *E. cloacae* ATCC 13047 as references. Natural missense mutations identified in classical chromosomal genes previously associated with β-lactam resistance (e.g., *mrdA*, *ftsI*, *ramA*, *marA*, *acrR*, and *tolC*) were reviewed and filtered out after screening a representative collection of CPE with a wild-type antimicrobial susceptibility profile toward identifying new β-lactam/β-lactamase inhibitor combinations.

### Antimicrobial susceptibility testing

MICs of meropenem, meropenem/xeruborbactam, meropenem/vaborbactam, imipenem, imipenem/relebactam, cefepime, cefepime/taniborbactam, ceftazidime, ceftazidime/avibactam, aztreonam, and aztreonam/avibactam were determined in triplicate by reference broth microdilution assays with cation-adjusted Müeller-Hinton broth prepared according to Clinical and Laboratory Standards Institute (CLSI) M100 guidelines (51). Except for meropenem/xeruborbactam, the MICs of the β-lactam partner for the different β-lactam/β-lactamase inhibitor combinations are not shown throughout the results for conciseness but are provided in Tables S3 to S5. MIC values were interpreted following European Committee on Antimicrobial Susceptibility Testing (EUCAST) guidelines (v.14.0) for Enterobacterales (https://www.eucast.org/clinical_breakpoints). Clinical breakpoints for combinations that have not yet been approved were interpreted using those of the β-lactam partner. Avibactam, relebactam, and taniborbactam were tested at a fixed concentration of 4 mg/L, while vaborbactam and xeruborbactam were tested at 8 mg/L. Reference strains *E. coli* ATCC 25922, *K. pneumoniae* ATCC 700603, *E. coli* NCTC 13353, and *Acinetobacter baumannii* NCTC 13304 (for cephalosporin/β-lactamase inhibitor combinations), and *K. pneumoniae* ATCC BAA-2814 (for carbapenem/β-lactamase inhibitor combinations) were used as controls for comparative purposes.

## SUPPLEMENTAL MATERIAL

Supplemental material aac.00533-25-s0001.docx Tables S1 to S5.

ASM does not own the copyrights to Supplemental Material that may be linked to, or accessed through, an article. The authors have granted ASM a non-exclusive, world-wide license to publish the Supplemental Material files. Please contact the corresponding author directly for reuse.
