# Supplementary material for: Broad spectrum of β-lactamase coverage and potent antimicrobial activity of xeruborbactam in combination with meropenem against carbapenemase-producing Enterobacterales, including strains resistant to new β-lactam/β-lactamase inhibitor combinations
Source: Antimicrob Agents Chemother. 2025 Jul 25;69(9):e00533-25. doi: 10.1128/aac.00533-25 (PMC12406686; doi:10.1128/aac.00533-25)
Supplement: Supplemental material — Tables S1 to S5. [file aac.00533-25-s0001.docx]

| **Table S1.** Distribution of the isolates studied according to bacterial species. | | | | |
| --- | --- | --- | --- | --- |
| **Bacterial species** | **All** | **OXA-48-like** | **KPC-like** | **MBL** |
|  | **(n=300)** | **(n=100)** | **(n=100)** | **(n=100)** |
| *Citrobacter freundii* | 16 | 4 | 7 | 5 |
| *Enterobacter cloacae* complex | 55 | 18 | 8 | 29 |
| *Escherichia coli* | 40 | 17 | 9 | 14 |
| *Klebsiella aerogenes* | 2 | - | - | 2 |
| *Klebsiella oxytoca* | 19 | 5 | 4 | 10 |
| *Klebsiella pneumoniae* | 166 | 56 | 72 | 38 |
| *Providencia rettgeri* | 1 | - | - | 1 |
| *Raoultella planticola* | 1 | - | - | 1 |

| **Table S2.** Distribution of isolates according to the type of carbapenemase produced. | | | | | | | | |
| --- | --- | --- | --- | --- | --- | --- | --- | --- |
| **OXA-48-like (n=100)** | |  | **KPC-like (n=100)** | | |  | **MBL (n=100)** | |
| OXA-48 | 96 |  | KPC-2 | | 42 |  | IMP-8 | 4 |
| OXA-181 | 2 |  | KPC-3 | | 54 |  | IMP-22 | 1 |
| OXA-244 | 2 |  | KPC-23 | | 1 |  | NDM-1 | 20 |
|  |  |  | KPC-31 | | 1 |  | NDM-5 | 6 |
|  |  |  | KPC-66 | | 1 |  | NDM-7 | 4 |
|  |  |  | KPC-132 | | 1 |  | NDM-14 | 1 |
|  |  |  |  | |  |  | NDM-23 | 1 |
|  |  |  |  | |  |  | VIM-1 | 61 |
|  |  | |  |  |  |  | VIM-4 | 1 |
|  |  | |  |  |  |  | VIM-23 | 1 |

| **Table S3.** Antimicrobial susceptibility data for meropenem/xeruborbactam compared to other newly developed β-lactamase inhibitor/β-lactam combinations against OXA-48-like-producing Enterobacterales (n=100). | | | | | | | | | | | | | | | | |
| --- | --- | --- | --- | --- | --- | --- | --- | --- | --- | --- | --- | --- | --- | --- | --- | --- |
| **BioProject ID** | **Genome number** | **Species** | **Hospital code** | **MLST** | **Carbapenemase** | **MIC (mg/L)^a^** | | | | | | | | | | |
|  |  |  |  |  |  | **MEM**  **(R>8)** | **M/X**  **(R>8)** | **M/V**  **(R>8)** | **IMP**  **(R>4)** | **I/R**  **(R>2)** | **FEP**  **(R>4)** | **F/T**  **(R>4)** | **CAZ**  **(R>4)** | **C/A**  **(R>8)** | **ATM**  **(R>4)** | **A/A**  **(R>4)** |
| PRJEB42440 | AI2595 | *Klebsiella pneumoniae* | MAD01 | 405 | OXA-48 | 16 | ≤0.06 | 16 | 8 | 2 | >64 | 1 | >64 | 4 | >64 | 0.125 |
| PRJEB42440 | AI2600 | *Klebsiella pneumoniae* | MAD01 | 405 | OXA-48 | 16 | ≤0.06 | 16 | 8 | 4 | >64 | 4 | >64 | 4 | >64 | 0.125 |
| PRJEB42440 | AI2604 | *Klebsiella pneumoniae* | MAD01 | 11 | OXA-48 | 0.25 | ≤0.06 | 0.125 | 1 | 0.5 | >64 | 0.25 | >64 | 4 | >64 | 0.125 |
| PRJEB42440 | AI2838 | *Klebsiella pneumoniae* | CAT01 | 307 | OXA-48 | 64 | 0.125 | 64 | >64 | 64 | >64 | 8 | >64 | 1 | >64 | 0.125 |
| PRJEB42440 | AH0326 | *Klebsiella pneumoniae* | CAT01 | 392 | OXA-48 | 16 | ≤0.06 | 16 | 16 | 8 | >64 | 2 | 1 | 1 | >64 | 0.125 |
| PRJEB42440 | AI2847 | *Klebsiella pneumoniae* | CAT01 | 307 | OXA-48 | 64 | 0.125 | 64 | 32 | 32 | >64 | 2 | >64 | 2 | >64 | 0.25 |
| PRJEB42440 | AH0327 | *Klebsiella pneumoniae* | CAT01 | 392 | OXA-48 | 1 | ≤0.06 | 1 | 4 | 4 | >64 | 0.25 | >64 | 1 | >64 | 0.125 |
| PRJEB42440 | AI2849 | *Klebsiella pneumoniae* | CAT02 | 147 | OXA-48 | 16 | ≤0.06 | 16 | 8 | 4 | >64 | 4 | >64 | 1 | >64 | 0.25 |
| PRJEB42440 | AI2852 | *Escherichia coli* | CAT02 | 10 | OXA-48 | 32 | 0.125 | 32 | 64 | 32 | >64 | 0.25 | >64 | 1 | 8 | 0.125 |
| PRJEB39112 | AI2855 | *Klebsiella pneumoniae* | CAT02 | 147 | OXA-48 | 32 | ≤0.06 | 16 | 16 | 8 | >64 | 2 | >64 | 4 | >64 | 0.125 |
| PRJEB39112 | AI2856 | *Klebsiella pneumoniae* | CAT02 | 147 | OXA-48 | 16 | ≤0.06 | 8 | 4 | 2 | >64 | 4 | >64 | 1 | >64 | 0.25 |
| PRJEB42440 | AI2866 | *Enterobacter cloacae* complex | MAD02 | 90 | OXA-48 | 1 | ≤0.06 | 1 | 2 | 2 | 0.5 | 0.125 | 4 | 1 | 0.125 | ≤0.06 |
| PRJEB42440 | AI2870 | *Klebsiella pneumoniae* | MAD03 | 11 | OXA-48 | 8 | ≤0.06 | 4 | 4 | 2 | >64 | 4 | >64 | 2 | >64 | 0.25 |
| PRJEB42440 | AI2879 | *Klebsiella pneumoniae* | MAD03 | 11 | OXA-48 | 1 | ≤0.06 | 1 | 16 | 16 | >64 | 2 | >64 | >64 | >64 | 0.125 |
| PRJEB42440 | AI2882 | *Klebsiella pneumoniae* | MAD03 | 307 | OXA-48 | 2 | ≤0.06 | 1 | 4 | 2 | 1 | 1 | >64 | 1 | 0.25 | 0.125 |
| PRJEB42440 | AI2890 | *Escherichia coli* | MAD03 | 538 | OXA-48 | 2 | ≤0.06 | 0.5 | 4 | 2 | 0.5 | 0.125 | 2 | 0.5 | 0.125 | ≤0.06 |
| PRJEB42440 | AI2893 | *Klebsiella pneumoniae* | MAD03 | 45 | OXA-48 | 0.25 | ≤0.06 | 0.25 | 0.5 | 0.5 | 0.5 | 0.125 | 0.5 | 0.125 | 0.125 | ≤0.06 |
| PRJEB42440 | AI2894 | *Escherichia coli* | MAD03 | 58 | OXA-48 | 0.25 | ≤0.06 | 0.25 | 1 | 0.5 | 0.5 | ≤0.06 | 0.5 | 0.125 | 0.125 | ≤0.06 |
| PRJEB42440 | AH0329 | *Klebsiella pneumoniae* | MAD04 | 392 | OXA-48 | 0.25 | ≤0.06 | 0.25 | 16 | 16 | 0.5 | 0.25 | >64 | 1 | 0.25 | 0.125 |
| PRJEB42440 | AI2926 | *Klebsiella pneumoniae* | MAD04 | 11 | OXA-48 | 0.5 | ≤0.06 | 0.5 | 2 | 2 | 32 | 0.5 | >64 | 0.5 | 64 | 0.125 |
| PRJEB42440 | AI2938 | *Escherichia coli* | BAL01 | 127 | OXA-48 | 0.5 | ≤0.06 | 0.25 | 2 | 1 | >64 | 0.25 | 32 | 0.25 | >64 | 0.125 |
| PRJEB42440 | AI2697 | *Enterobacter cloacae* complex | AST01 | 171 | OXA-48 | 0.5 | ≤0.06 | 0.5 | 1 | 1 | 64 | ≤0.06 | >64 | 0.5 | 64 | 0.25 |
| PRJEB42440 | AI2661 | *Citrobacter freundii* | AST01 | 22 | OXA-48 | 0.5 | ≤0.06 | 0.5 | 2 | 1 | 2 | 1 | >64 | 0.25 | >64 | 0.125 |
| PRJEB42440 | AI2662 | *Enterobacter cloacae* complex | AST01 | 171 | OXA-48 | 64 | 0.25 | 64 | >64 | >64 | >64 | 4 | >64 | 0.5 | >64 | 0.25 |
| PRJEB42440 | AI2666 | *Citrobacter freundii* | AST01 | 18 | OXA-48 | 0.5 | ≤0.06 | 1 | 1 | 1 | 16 | 0.5 | >64 | 0.5 | >64 | 0.25 |
| PRJEB42440 | AI2679 | *Enterobacter cloacae* complex | AST01 | 23 | OXA-48 | 1 | ≤0.06 | 1 | 2 | 1 | 0.5 | 0.25 | 2 | 0.5 | 0.5 | 0.25 |
| PRJEB42440 | AI2683 | *Enterobacter cloacae* complex | AST01 | 66 | OXA-48 | 0.5 | ≤0.06 | 0.5 | 1 | 0.5 | 64 | 1 | >64 | 2 | >64 | 0.125 |
| PRJEB42440 | AI2686 | *Klebsiella pneumoniae* | AST01 | 326 | OXA-48 | 2 | ≤0.06 | 2 | 4 | 4 | >64 | 2 | >64 | 0.5 | >64 | 0.125 |
| PRJEB42440 | AH0330 | *Klebsiella pneumoniae* | AST01 | 15 | OXA-48 | 0.5 | ≤0.06 | 0.5 | 32 | 32 | 64 | 0.25 | >64 | 0.5 | 32 | ≤0.06 |
| PRJEB42440 | AI2951 | *Escherichia coli* | AST01 | 131 | OXA-48 | 0.125 | ≤0.06 | 0.125 | 0.5 | 0.5 | 0.5 | ≤0.06 | 0.5 | 0.25 | ≤0.06 | ≤0.06 |
| PRJEB42440 | AI2693 | *Klebsiella pneumoniae* | AST01 | 567 | OXA-48 | 0.25 | ≤0.06 | 0.25 | 0.5 | 0.5 | 0.5 | ≤0.06 | 0.125 | ≤0.06 | ≤0.06 | ≤0.06 |
| PRJEB42440 | AI2695 | *Enterobacter cloacae* complex | AST01 | 78 | OXA-48 | 1 | ≤0.06 | 2 | 1 | 1 | 1 | 0.125 | 4 | 0.5 | 0.25 | ≤0.06 |
| PRJEB42440 | AI2952 | *Klebsiella pneumoniae* | AST01 | 326 | OXA-48 | 0.5 | ≤0.06 | 0.25 | 8 | 2 | 0.25 | ≤0.06 | 0.25 | 0.125 | 64 | ≤0.06 |
| PRJEB42440 | AI2959 | *Escherichia coli* | CLM01 | 68 | OXA-48 | 0.5 | ≤0.06 | 0.5 | 2 | 2 | 32 | 0.5 | 2 | 1 | 4 | ≤0.06 |
| PRJEB42440 | AI2962 | *Klebsiella pneumoniae* | CLM01 | 307 | OXA-48 | 64 | 0.125 | 64 | >64 | >64 | >64 | 4 | >64 | 0.5 | >64 | 0.25 |
| PRJEB42440 | AI2966 | *Klebsiella pneumoniae* | CLM01 | 307 | OXA-48 | 1 | ≤0.06 | 0.5 | 2 | 1 | 64 | 0.25 | >64 | 32 | >64 | ≤0.06 |
| PRJEB42440 | AI2967 | *Klebsiella pneumoniae* | CLM01 | 405 | OXA-48 | 1 | ≤0.06 | 1 | 2 | 2 | 1 | 0.25 | >64 | 0.5 | ≤0.06 | ≤0.06 |
| PRJEB42440 | AH0331 | *Klebsiella pneumoniae* | CLM01 | 104 | OXA-48 | 32 | 0.125 | 32 | 32 | 32 | 2 | 1 | 1 | 0.5 | 0.25 | 0.125 |
| PRJEB42440 | AI2908 | *Klebsiella pneumoniae* | MAD04 | 39 | OXA-48 | 0.5 | ≤0.06 | 0.5 | 1 | 1 | >64 | 2 | >64 | 2 | >64 | ≤0.06 |
| PRJEB39112 | AI2766 | *Klebsiella pneumoniae* | CAT03 | 147 | OXA-48 | 64 | 0.25 | 64 | 64 | 32 | >64 | 4 | >64 | 2 | >64 | 0.5 |
| PRJEB39112 | AI2767 | *Klebsiella pneumoniae* | CAT03 | 147 | OXA-48 | 16 | ≤0.06 | 16 | 2 | 2 | >64 | 1 | >64 | 1 | >64 | 0.5 |
| PRJEB39112 | AI2993 | *Klebsiella pneumoniae* | CAT03 | 147 | OXA-48 | 16 | ≤0.06 | 16 | 2 | 2 | >64 | 2 | >64 | 2 | >64 | 0.125 |
| PRJEB39112 | AI2768 | *Klebsiella pneumoniae* | CAT03 | 147 | OXA-48 | 16 | ≤0.06 | 16 | 4 | 2 | >64 | 2 | >64 | 0.5 | >64 | 0.25 |
| PRJEB39112 | AI2996 | *Klebsiella pneumoniae* | CAT03 | 147 | OXA-48 | 16 | ≤0.06 | 16 | 2 | 1 | >64 | 2 | >64 | 2 | >64 | 0.125 |
| PRJEB39112 | AI2998 | *Klebsiella pneumoniae* | CAT03 | 147 | OXA-48 | 32 | 0.125 | 16 | 2 | 0.5 | >64 | 1 | >64 | 1 | >64 | 0.125 |
| PRJEB42440 | AN2336 | *Klebsiella oxytoca* | AST01 | 27 | OXA-48 | 0.5 | ≤0.06 | 0.5 | 2 | 1 | 4 | 1 | 2 | 0.5 | 0.125 | 0.125 |
| PRJEB42440 | AI2787 | *Klebsiella pneumoniae* | VAL01 | 307 | OXA-48 | 1 | ≤0.06 | 1 | 2 | 1 | 32 | 1 | >64 | 1 | 32 | ≤0.06 |
| PRJEB42440 | AI3011 | *Klebsiella pneumoniae* | CAT04 | 13 | OXA-48 | 8 | ≤0.06 | 2 | 2 | 1 | 4 | 0.25 | 2 | 1 | 0.25 | ≤0.06 |
| PRJEB42440 | AI2801 | *Klebsiella pneumoniae* | CAT04 | 101 | OXA-48 | 32 | ≤0.06 | 32 | 4 | 2 | >64 | 2 | >64 | 4 | >64 | 0.125 |
| PRJEB42440 | AI2935 | *Citrobacter freundii* | AND02 | 112 | OXA-48 | 1 | ≤0.06 | 0.5 | 2 | 0.5 | >64 | 0.25 | 64 | 0.5 | 8 | 0.125 |
| PRJEB42440 | AI2624 | *Enterobacter cloacae* complex | VAL02 | 114 | OXA-48 | 16 | ≤0.06 | 4 | 1 | 1 | 32 | 4 | >64 | 2 | >64 | 2 |
| PRJEB42440 | AI2915 | *Enterobacter cloacae* complex | MAD04 | 114 | OXA-48 | 64 | 0.125 | 64 | 8 | 8 | 4 | 1 | >64 | 4 | 8 | 0.25 |
| PRJEB42440 | AI2949 | *Escherichia coli* | AST01 | 131 | OXA-48 | 0.5 | ≤0.06 | 0.25 | 1 | 0.5 | 4 | 1 | 1 | 0.125 | 0.125 | ≤0.06 |
| PRJEB42440 | AI2623 | *Klebsiella oxytoca* | VAL02 | 145 | OXA-48 | 2 | ≤0.06 | 4 | 2 | 2 | >64 | 1 | >64 | 0.5 | >64 | 0.25 |
| PRJEB42440 | AI2850 | *Klebsiella pneumoniae* | CAT02 | 151 | OXA-48 | 0.125 | ≤0.06 | 0.125 | 1 | 1 | 0.125 | ≤0.06 | ≤0.06 | ≤0.06 | ≤0.06 | ≤0.06 |
| PRJEB42440 | AI2851 | *Klebsiella pneumoniae* | CAT02 | 152 | OXA-48 | 1 | ≤0.06 | 1 | 4 | 2 | >64 | 1 | >64 | 4 | >64 | 0.125 |
| PRJEB42440 | AI2854 | *Klebsiella pneumoniae* | CAT02 | 152 | OXA-48 | 1 | ≤0.06 | 1 | 2 | 1 | >64 | 1 | >64 | 2 | >64 | 0.125 |
| PRJEB42440 | AN2364 | *Enterobacter cloacae* complex | GAL02 | 168 | OXA-48 | 1 | ≤0.06 | 1 | 0.5 | 0.5 | 4 | 1 | >64 | 1 | 8 | 0.25 |
| PRJEB42440 | AH0328 | *Enterobacter cloacae* complex | BAL01 | 171 | OXA-48 | 0.5 | ≤0.06 | 0.5 | 2 | 2 | 2 | 1 | 1 | 1 | 0.25 | ≤0.06 |
| PRJEB42440 | AI2844 | *Klebsiella pneumoniae* | CAT01 | 198 | OXA-48 | 0.5 | ≤0.06 | 0.5 | 1 | 0.5 | 0.25 | ≤0.06 | 0.25 | 0.125 | 0.25 | ≤0.06 |
| PRJEB42440 | AI2593 | *Klebsiella pneumoniae* | MAD01 | 219 | OXA-48 | 0.5 | ≤0.06 | 0.5 | 0.5 | 0.5 | 64 | 1 | 64 | 0.5 | 64 | 0.125 |
| PRJEB42440 | AI2699 | *Klebsiella oxytoca* | AST01 | 325 | OXA-48 | 0.5 | ≤0.06 | 0.25 | 1 | 1 | 64 | ≤0.06 | >64 | 0.25 | 32 | ≤0.06 |
| PRJEB42440 | AI2943 | *Klebsiella oxytoca* | AST01 | 327 | OXA-48 | 1 | ≤0.06 | 1 | 2 | 2 | 16 | 8 | 1 | 0.25 | 0.25 | ≤0.06 |
| PRJEB42440 | AI2637 | *Klebsiella pneumoniae* | CAT01 | 429 | OXA-48 | 0.5 | ≤0.06 | 0.5 | 0.25 | 0.25 | >64 | 0.25 | >64 | 1 | >64 | ≤0.06 |
| PRJEB42440 | AI2792 | *Klebsiella pneumoniae* | VAL01 | 437 | OXA-48 | 1 | ≤0.06 | 1 | 0.5 | 0.5 | 32 | 4 | >64 | 1 | >64 | 0.5 |
| PRJEB42440 | AI2667 | *Klebsiella pneumoniae* | AST01 | 485 | OXA-48 | 0.5 | ≤0.06 | 0.5 | 2 | 1 | 0.25 | ≤0.06 | 0.5 | 0.125 | 0.25 | ≤0.06 |
| PRJEB42440 | AN2346 | *Klebsiella pneumoniae* | GAL02 | 551 | OXA-48 | 2 | ≤0.06 | 1 | 2 | 1 | 1 | ≤0.06 | 0.5 | 0.125 | 0.25 | ≤0.06 |
| PRJEB42440 | AI2834 | *Escherichia coli* | VAL02 | 624 | OXA-48 | 1 | ≤0.06 | 1 | 1 | 0.5 | 2 | 1 | 4 | 1 | 0.5 | 0.5 |
| PRJEB42440 | AI2803 | *Klebsiella pneumoniae* | CAT04 | 628 | OXA-48 | 2 | ≤0.06 | 2 | 2 | 2 | 1 | 0.25 | 2 | 0.5 | 0.25 | ≤0.06 |
| PRJEB42440 | AI2904 | *Enterobacter cloacae* complex | MAD04 | 662 | OXA-48 | 0.25 | ≤0.06 | 0.25 | 2 | 2 | >64 | 0.5 | >64 | 2 | >64 | 0.25 |
| PRJEB42440 | AI2905 | *Enterobacter cloacae* complex | MAD04 | 732 | OXA-48 | 2 | ≤0.06 | 1 | 1 | 1 | >64 | 0.5 | 64 | 0.5 | >64 | 0.25 |
| PRJEB42440 | AI2672 | *Enterobacter cloacae* complex | AST01 | 1379 | OXA-48 | 4 | ≤0.06 | 4 | 1 | 1 | 64 | 1 | >64 | 2 | >64 | 2 |
| PRJEB42440 | AI2796 | *Enterobacter cloacae* complex | CAT04 | 1380 | OXA-48 | 1 | ≤0.06 | 0.5 | 0.5 | 0.5 | 4 | 0.5 | >64 | 1 | >64 | 1 |
| PRJEB42440 | AI2999 | *Enterobacter cloacae* complex | CAT03 | 1382 | OXA-48 | 0.5 | ≤0.06 | 0.5 | 1 | 0.5 | 0.25 | ≤0.06 | 32 | 0.5 | 0.25 | 0.125 |
| PRJEB42440 | AI2842 | *Klebsiella pneumoniae* | CAT01 | 1401 | OXA-48 | 0.25 | ≤0.06 | 0.25 | 1 | 0.5 | 0.25 | ≤0.06 | 0.5 | 0.5 | 0.25 | ≤0.06 |
| PRJEB42440 | AI3027 | *Klebsiella pneumoniae* | AND01 | 3362 | OXA-48 | 1 | ≤0.06 | 1 | 4 | 4 | >64 | 1 | 32 | 1 | >64 | 0.125 |
| PRJEB42440 | AI3034 | *Klebsiella pneumoniae* | AST01 | 4387 | OXA-48 | 0.125 | ≤0.06 | ≤0.06 | 0.25 | 0.25 | 0.125 | ≤0.06 | 0.25 | 0.125 | 0.125 | ≤0.06 |
| PRJEB42440 | AI2740 | *Klebsiella pneumoniae* | CAN01 | 5000 | OXA-48 | 0.5 | ≤0.06 | 0.5 | 1 | 0.5 | >64 | ≤0.06 | >64 | 2 | 64 | ≤0.06 |
| PRJEB42440 | AI2907 | *Klebsiella pneumoniae* | MAD04 | 5001 | OXA-48 | 0.5 | ≤0.06 | 0.5 | 2 | 1 | 64 | 0.25 | >64 | 1 | 64 | ≤0.06 |
| PRJEB42440 | AI2974 | *Klebsiella pneumoniae* | CLM01 | 5002 | OXA-48 | 0.5 | ≤0.06 | 0.5 | 2 | 2 | 16 | 0.125 | 64 | 0.5 | >64 | ≤0.06 |
| PRJEB42440 | AI2994 | *Escherichia coli* | CAT03 | 11106 | OXA-48 | 0.25 | ≤0.06 | 0.25 | 0.5 | 0.5 | 0.5 | ≤0.06 | >64 | 0.5 | 0.25 | ≤0.06 |
| PRJEB53700 | 20200560 | *Escherichia coli* | AND15 | 1049 | OXA-48 | ≤0.06 | ≤0.06 | ≤0.06 | 1 | 0.25 | 0.125 | ≤0.06 | 1 | ≤0.06 | ≤0.06 | ≤0.06 |
| PRJEB53700 | 20211130 | *Klebsiella oxytoca* | AND07 | 108 | OXA-48 | 2 | ≤0.06 | 2 | 1 | 0.5 | 0.5 | ≤0.06 | 1 | 0.25 | 0.5 | 0.125 |
| PRJEB53700 | 20220523 | *Enterobacter cloacae* complex | AND17 | 110 | OXA-48 | 1 | ≤0.06 | 1 | 2 | 1 | 8 | ≤0.06 | 64 | 1 | 4 | 0.125 |
| PRJEB53700 | 20200300 | *Escherichia coli* | AND02 | 11249 | OXA-48 | 0.25 | ≤0.06 | 0.25 | 1 | 0.5 | 32 | 0.125 | 32 | 0.25 | 32 | ≤0.06 |
| PRJEB53700 | 20190736 | *Escherichia coli* | AND07 | 1193 | OXA-48 | 0.25 | ≤0.06 | 0.25 | 0.5 | 0.25 | 0.25 | ≤0.06 | 0.125 | ≤0.06 | ≤0.06 | ≤0.06 |
| PRJEB53700 | 20190666 | *Enterobacter cloacae* complex | AND07 | 120 | OXA-48 | 1 | ≤0.06 | 1 | 1 | 1 | 1 | 0.25 | 1 | 0.5 | 0.125 | 0.125 |
| PRJEB53700 | 20200049 | *Citrobacter freundii* | AND01 | 125 | OXA-48 | 1 | ≤0.06 | 1 | 0.5 | 0.25 | 4 | 1 | >64 | 0.5 | 32 | 0.25 |
| PRJEB53700 | 20190392 | *Klebsiella pneumoniae* | AND07 | 1411 | OXA-48 | 1 | ≤0.06 | 1 | 1 | 1 | 1 | ≤0.06 | 4 | 0.25 | 0.25 | 0.125 |
| PRJEB53700 | 20190733 | *Klebsiella pneumoniae* | AND07 | 1563 | OXA-48 | 1 | ≤0.06 | 0.5 | 1 | 0.5 | 0.25 | ≤0.06 | 0.5 | 0.25 | 0.125 | ≤0.06 |
| PRJEB53700 | 20200182 | *Klebsiella pneumoniae* | AND07 | 1565 | OXA-48 | 0.25 | ≤0.06 | 0.25 | 1 | 0.5 | 0.125 | ≤0.06 | 0.25 | ≤0.06 | ≤0.06 | ≤0.06 |
| PRJEB53700 | 20200206 | *Escherichia coli* | AND07 | 1582 | OXA-48 | 0.25 | ≤0.06 | 0.25 | 0.5 | 0.25 | 0.125 | ≤0.06 | 0.125 | ≤0.06 | ≤0.06 | ≤0.06 |
| PRJEB53700 | 20210142 | *Enterobacter cloacae* complex | AND07 | 1599 | OXA-48 | 2 | ≤0.06 | 2 | 1 | 1 | 64 | 1 | >64 | 1 | >64 | 0.25 |
| PRJEB53700 | 20200179 | *Klebsiella pneumoniae* | AND07 | 17 | OXA-48 | 0.25 | ≤0.06 | 0.25 | 2 | 0.5 | 0.5 | ≤0.06 | 0.25 | 0.125 | ≤0.06 | ≤0.06 |
| PRJEB53700 | 20210183 | *Escherichia coli* | AND11 | 58 | OXA-48 | 0.125 | ≤0.06 | ≤0.06 | 0.25 | 0.125 | 0.125 | ≤0.06 | ≤0.06 | ≤0.06 | ≤0.06 | ≤0.06 |
| PRJEB53700 | 20220475 | *Klebsiella pneumoniae* | AND18 | 23 | OXA-48 | 8 | ≤0.06 | 8 | 4 | 2 | >64 | 1 | >64 | 2 | >64 | 0.125 |
| PRJEB53700 | 20220622 | *Escherichia coli* | AND16 | 10 | OXA-244 | ≤0.06 | ≤0.06 | ≤0.06 | 0.125 | ≤0.06 | 0.125 | ≤0.06 | 32 | 0.25 | 4 | ≤0.06 |
| PRJEB53700 | 20220203 | *Escherichia coli* | AND08 | 1722 | OXA-244 | 0.5 | ≤0.06 | 0.5 | 0.125 | 0.125 | 1 | 0.5 | 1 | 0.125 | 0.25 | ≤0.06 |
| PRJEB53700 | 20220042 | *Klebsiella pneumoniae* | AND02 | 1758 | OXA-181 | 0.25 | ≤0.06 | 0.25 | 0.5 | 0.125 | 0.25 | ≤0.06 | >64 | 0.125 | ≤0.06 | ≤0.06 |
| PRJEB53700 | 20220160 | *Escherichia coli* | AND11 | 2083 | OXA-181 | 16 | 0.5 | 16 | 8 | 8 | >64 | 32 | >64 | 16 | 64 | 16 |
| MEM: meropenem; M/X: meropenem/xeruborbactam; M/V: meropenem/vaborbactam; IMP: imipenem; I/R: imipenem/relebactam; FEP: cefepime; F/T: cefepime/taniborbactam; CAZ: ceftazidime; C/A: ceftazidime/avibactam; ATM: aztreonam; A/A: aztreonam/avibactam.  ^a^ EUCAST breakpoints indicated for Enterobacterales. Clinical breakpoints for combinations that have not yet approved (meropenem/xeruborbactam and cefepime/taniborbactam) were interpreted using those of the β-lactam partner. Avibactam, relebactam, and taniborbactam were tested at a fixed concentration of 4 mg/L, while vaborbactam and xeruborbactam were tested at 8 mg/L. | | | | | | | | | | | | | | | | |

| **Table S4.** Antimicrobial susceptibility data for meropenem/xeruborbactam compared to other newly developed β-lactamase inhibitor/β-lactam combinations against KPC-like-producing Enterobacterales (n=100). | | | | | | | | | | | | | | | | |
| --- | --- | --- | --- | --- | --- | --- | --- | --- | --- | --- | --- | --- | --- | --- | --- | --- |
| **BioProject ID** | **Strain number** | **Species** | **Hospital code** | **MLST** | **Carbapenemase** | **MIC (mg/L)^a^** | | | | | | | | | | |
|  |  |  |  |  |  | **MEM**  **(R>8)** | **M/X**  **(R>8)** | **M/V**  **(R>8)** | **IMP**  **(R>4)** | **I/R**  **(R>2)** | **FEP**  **(R>4)** | **F/T**  **(R>4)** | **CAZ**  **(R>4)** | **C/A**  **(R>8)** | **ATM**  **(R>4)** | **A/A**  **(R>4)** |
| PRJEB42440 | AI2602 | *Citrobacter freundii* | MAD01 | 112 | KPC-2 | 16 | ≤0.06 | ≤0.06 | 4 | ≤0.06 | >64 | 1 | >64 | 1 | >64 | ≤0.06 |
| PRJEB42440 | AI2826 | *Citrobacter freundii* | MAD01 | 22 | KPC-2 | 8 | ≤0.06 | ≤0.06 | 8 | ≤0.06 | >64 | 0.25 | >64 | 1 | >64 | 0.125 |
| PRJEB42440 | AI2614 | *Klebsiella oxytoca* | CYL01 | 324 | KPC-2 | 2 | ≤0.06 | ≤0.06 | 2 | 0.25 | 32 | 0.5 | >64 | 2 | >64 | ≤0.06 |
| PRJEB53700 | 20220595 | *Klebsiella oxytoca* | AND18 | 108 | KPC-2 | 2 | ≤0.06 | ≤0.06 | 4 | 0.125 | 2 | ≤0.06 | >64 | 1 | 16 | ≤0.06 |
| PRJEB53700 | 20190510 | *Klebsiella pneumoniae* | AND09 | 307 | KPC-2 | 0.5 | ≤0.06 | ≤0.06 | 1 | ≤0.06 | >64 | 0.5 | >64 | 2 | >64 | 0.125 |
| PRJEB53700 | 20190576 | *Citrobacter freundii* | AND06 | 8 | KPC-2 | 2 | ≤0.06 | ≤0.06 | 2 | ≤0.06 | 32 | 0.25 | >64 | 0.5 | >64 | 0.125 |
| PRJEB53700 | 20190661 | *Enterobacter cloacae* complex | AND03 | 350 | KPC-2 | 1 | ≤0.06 | ≤0.06 | 2 | 0.125 | 8 | ≤0.06 | 8 | 0.25 | 32 | ≤0.06 |
| PRJEB53700 | 20190771 | *Klebsiella pneumoniae* | AND08 | 4973 | KPC-2 | 2 | ≤0.06 | ≤0.06 | 0.5 | ≤0.06 | 8 | ≤0.06 | 64 | 0.5 | >64 | 0.25 |
| PRJEB53700 | 20200474 | *Klebsiella pneumoniae* | AND17 | 15 | KPC-2 | 8 | ≤0.06 | ≤0.06 | 1 | ≤0.06 | 16 | 0.125 | 64 | 2 | >64 | 0.125 |
| PRJEB53700 | 20200479 | *Escherichia coli* | AND02 | 357 | KPC-2 | 2 | ≤0.06 | ≤0.06 | 2 | 0.25 | 16 | 0.125 | 64 | 0.25 | >64 | ≤0.06 |
| PRJEB53700 | 20220247 | *Citrobacter freundii* | AND06 | 730 | KPC-2 | 1 | ≤0.06 | 0.5 | 1 | 0.5 | 2 | ≤0.06 | >64 | 0.5 | 16 | 0.125 |
| PRJEB53700 | 20210037 | *Escherichia coli* | AND17 | 648 | KPC-2 | 2 | ≤0.06 | ≤0.06 | 0.5 | 0.125 | 64 | ≤0.06 | 32 | 0.125 | 64 | ≤0.06 |
| PRJEB53700 | 20210342 | *Klebsiella oxytoca* | AND12 | 170 | KPC-2 | 1 | ≤0.06 | ≤0.06 | 2 | 0.25 | 1 | ≤0.06 | 16 | 0.5 | 64 | 0.125 |
| PRJEB53700 | 20200356 | *Klebsiella pneumoniae* | AND16 | 258 | KPC-2 | 32 | ≤0.06 | ≤0.06 | 32 | 0.25 | 32 | ≤0.06 | >64 | 2 | >64 | 0.125 |
| PRJEB53700 | 20210847 | *Escherichia coli* | AND18 | 327 | KPC-2 | 1 | ≤0.06 | ≤0.06 | 2 | 0.125 | 4 | ≤0.06 | >64 | 1 | 64 | ≤0.06 |
| PRJEB53700 | 20210607 | *Enterobacter cloacae* complex | AND13 | 93 | KPC-2 | 16 | ≤0.06 | ≤0.06 | 8 | ≤0.06 | >64 | 0.5 | >64 | 2 | >64 | 0.25 |
| PRJNA1133624 | ARGA00246 | *Klebsiella pneumoniae* | GAL02 | 307 | KPC-2 | 4 | ≤0.06 | ≤0.06 | 8 | 0.25 | >64 | 0.5 | >64 | 1 | >64 | 0.125 |
| PRJNA1133624 | ARGA00248 | *Escherichia coli* | GAL02 | 506 | KPC-2 | 0.5 | ≤0.06 | ≤0.06 | 1 | 0.125 | 2 | ≤0.06 | 2 | 0.125 | 16 | ≤0.06 |
| PRJNA1133624 | ARGA00260 | *Klebsiella pneumoniae* | GAL02 | 307 | KPC-2 | 8 | ≤0.06 | ≤0.06 | 8 | 0.25 | >64 | 0.125 | >64 | 2 | >64 | 0.125 |
| PRJNA1133624 | ARGA00262 | *Citrobacter freundii* | GAL02 | 155 | KPC-2 | >64 | 2 | 64 | >64 | 4 | >64 | 2 | >64 | 4 | >64 | 2 |
| PRJEB42440 | AI2700 | *Klebsiella pneumoniae* | GAL02 | 1961 | KPC-2 | 2 | ≤0.06 | ≤0.06 | 4 | 0.25 | 16 | ≤0.06 | 32 | 1 | >64 | ≤0.06 |
| PRJEB42440 | AI2701 | *Klebsiella pneumoniae* | GAL02 | 273 | KPC-2 | 64 | ≤0.06 | 0.25 | 32 | 0.125 | 64 | 0.5 | 32 | 1 | >64 | 0.125 |
| PRJEB42440 | AN2340 | *Klebsiella pneumoniae* | GAL02 | 2295 | KPC-2 | 1 | ≤0.06 | ≤0.06 | 2 | 1 | 2 | ≤0.06 | 4 | 0.125 | 16 | ≤0.06 |
| PRJEB42440 | AI2703 | *Escherichia coli* | GAL02 | 162 | KPC-2 | 8 | ≤0.06 | ≤0.06 | 4 | ≤0.06 | 32 | ≤0.06 | 32 | 0.5 | >64 | ≤0.06 |
| PRJEB42440 | AI2704 | *Enterobacter cloacae* complex | GAL02 | 96 | KPC-2 | 32 | ≤0.06 | 0.125 | 32 | 0.5 | >64 | 0.5 | >64 | 2 | >64 | 0.5 |
| PRJEB42440 | AI2979 | *Klebsiella pneumoniae* | GAL02 | 1961 | KPC-2 | 2 | ≤0.06 | 0.25 | 4 | ≤0.06 | 16 | 0.125 | 32 | 0.5 | >64 | 0.125 |
| PRJEB42440 | AI2705 | *Enterobacter cloacae* complex | GAL02 | 515 | KPC-2 | 16 | ≤0.06 | 0.125 | 8 | 0.5 | 16 | 0.125 | 64 | 0.5 | >64 | 0.5 |
| PRJNA1133624 | ARGA00426 | *Citrobacter freundii* | GAL02 | 85 | KPC-2 | 64 | ≤0.06 | 0.125 | 64 | 0.5 | 64 | 0.5 | 32 | 0.5 | >64 | 0.25 |
| PRJEB42440 | AI2710 | *Enterobacter cloacae* complex | GAL02 | 96 | KPC-2 | 64 | ≤0.06 | 0.25 | 32 | 0.5 | >64 | 1 | >64 | 2 | >64 | 0.5 |
| PRJEB42440 | AI2711 | *Klebsiella oxytoca* | GAL02 | N/A | KPC-2 | 8 | ≤0.06 | ≤0.06 | 4 | 0.5 | 16 | ≤0.06 | 8 | 1 | >64 | 0.125 |
| PRJEB42440 | AI2980 | *Klebsiella pneumoniae* | GAL02 | 1961 | KPC-2 | 2 | ≤0.06 | ≤0.06 | 2 | 0.25 | 16 | ≤0.06 | 16 | 1 | >64 | 0.125 |
| PRJEB42440 | AI2981 | *Escherichia coli* | GAL02 | 10 | KPC-2 | 0.125 | ≤0.06 | ≤0.06 | 1 | ≤0.06 | 2 | ≤0.06 | 8 | 0.125 | 32 | ≤0.06 |
| PRJEB42440 | AI2982 | *Klebsiella pneumoniae* | GAL02 | 1961 | KPC-2 | 4 | ≤0.06 | ≤0.06 | 2 | 0.125 | 16 | ≤0.06 | 64 | 0.5 | >64 | 0.125 |
| PRJEB42440 | AI2713 | *Klebsiella pneumoniae* | GAL02 | 273 | KPC-2 | >64 | ≤0.06 | 0.5 | 64 | 0.125 | >64 | 0.5 | 64 | 0.5 | >64 | 0.125 |
| PRJEB42440 | AI2715 | *Escherichia coli* | GAL02 | 23 | KPC-2 | 8 | ≤0.06 | ≤0.06 | 8 | 0.25 | 32 | ≤0.06 | 64 | 0.25 | >64 | ≤0.06 |
| PRJEB42440 | AI2983 | *Enterobacter cloacae* complex | GAL02 | 1381 | KPC-2 | 16 | ≤0.06 | ≤0.06 | 8 | 0.25 | 64 | ≤0.06 | 64 | 16 | >64 | ≤0.06 |
| PRJEB42440 | AI2717 | *Klebsiella pneumoniae* | GAL02 | 1961 | KPC-2 | 4 | ≤0.06 | ≤0.06 | 2 | 0.125 | 16 | ≤0.06 | 32 | 0.5 | >64 | 0.125 |
| PRJEB42440 | AI2984 | *Enterobacter cloacae* complex | GAL02 | 515 | KPC-2 | >64 | 0.25 | 2 | 64 | 1 | 32 | 0.25 | 16 | 0.5 | >64 | 0.25 |
| PRJEB42440 | AI2719 | *Enterobacter cloacae* complex | GAL02 | 515 | KPC-2 | 0.25 | ≤0.06 | 0.125 | 0.5 | 0.5 | 0.25 | 0.25 | 8 | 0.5 | 16 | 0.5 |
| PRJEB42440 | AI2985 | *Klebsiella pneumoniae* | GAL02 | 1961 | KPC-2 | 4 | ≤0.06 | 0.125 | 4 | 0.125 | 32 | 0.125 | 16 | 1 | >64 | 0.125 |
| PRJEB42440 | AI3060 | *Klebsiella pneumoniae* | GAL02 | 1961 | KPC-2 | 4 | ≤0.06 | ≤0.06 | 4 | ≤0.06 | >64 | 0.5 | 16 | 1 | >64 | 0.125 |
| PRJEB42440 | AI2721 | *Escherichia coli* | GAL02 | 131 | KPC-2 | 0.25 | ≤0.06 | ≤0.06 | 1 | ≤0.06 | 4 | 0.5 | 4 | ≤0.06 | 16 | ≤0.06 |
| PRJEB42440 | AI2588 | *Klebsiella pneumoniae* | MAD01 | 512 | KPC-3 | 16 | ≤0.06 | ≤0.06 | 4 | ≤0.06 | 32 | 0.25 | >64 | 4 | >64 | 0.125 |
| PRJEB42440 | AI2835 | *Klebsiella pneumoniae* | AND03 | 512 | KPC-3 | 64 | ≤0.06 | 0.25 | 16 | 0.125 | >64 | 1 | >64 | 4 | >64 | 0.125 |
| PRJEB42440 | AI2627 | *Klebsiella pneumoniae* | AND03 | 512 | KPC-3 | >64 | ≤0.06 | 0.25 | 32 | 0.125 | >64 | 1 | >64 | 16 | >64 | 0.125 |
| PRJEB42440 | AI2628 | *Klebsiella pneumoniae* | AND03 | 512 | KPC-3 | >64 | 0.125 | 0.5 | 16 | 0.125 | >64 | 2 | >64 | 8 | >64 | 0.125 |
| PRJEB42440 | AI2629 | *Klebsiella pneumoniae* | AND03 | 512 | KPC-3 | 32 | ≤0.06 | ≤0.06 | 4 | ≤0.06 | >64 | 2 | >64 | 4 | >64 | 0.125 |
| PRJEB42440 | AI2631 | *Klebsiella pneumoniae* | AND03 | 512 | KPC-3 | >64 | ≤0.06 | 0.5 | 16 | 0.125 | >64 | 2 | >64 | 16 | >64 | 0.125 |
| PRJEB42440 | AI2836 | *Klebsiella pneumoniae* | AND03 | 512 | KPC-3 | >64 | ≤0.06 | 1 | 16 | 0.125 | >64 | 1 | >64 | 8 | >64 | 0.125 |
| PRJEB42440 | AI2632 | *Klebsiella pneumoniae* | AND03 | 512 | KPC-3 | >64 | ≤0.06 | 0.5 | 8 | 0.125 | >64 | 1 | >64 | 8 | >64 | 0.125 |
| PRJEB42440 | AI2837 | *Klebsiella pneumoniae* | AND03 | 512 | KPC-3 | >64 | ≤0.06 | 0.5 | 32 | 0.125 | >64 | 2 | >64 | 8 | >64 | 0.125 |
| PRJEB42440 | AI2643 | *Klebsiella pneumoniae* | MAD03 | 307 | KPC-3 | 2 | ≤0.06 | ≤0.06 | 8 | 0.5 | 16 | 4 | >64 | 0.5 | >64 | ≤0.06 |
| PRJEB42440 | AI2644 | *Klebsiella pneumoniae* | MAD03 | 307 | KPC-3 | 2 | ≤0.06 | ≤0.06 | 2 | ≤0.06 | >64 | 1 | >64 | 2 | >64 | 0.125 |
| PRJEB42440 | AI2645 | *Klebsiella pneumoniae* | MAD03 | 307 | KPC-3 | 2 | ≤0.06 | ≤0.06 | 8 | 1 | 64 | 0.25 | >64 | 2 | 64 | 0.125 |
| PRJEB42440 | AI2883 | *Klebsiella pneumoniae* | MAD03 | 307 | KPC-3 | 4 | ≤0.06 | ≤0.06 | 16 | 1 | 64 | 4 | >64 | 0.5 | >64 | 0.125 |
| PRJEB42440 | AI2889 | *Klebsiella pneumoniae* | MAD03 | 307 | KPC-3 | 4 | ≤0.06 | ≤0.06 | 4 | 0.5 | 32 | 0.5 | >64 | 2 | >64 | 0.125 |
| PRJEB42440 | AI2646 | *Klebsiella pneumoniae* | MAD03 | 307 | KPC-3 | 4 | ≤0.06 | ≤0.06 | 8 | 1 | 64 | 0.5 | >64 | 1 | >64 | 0.125 |
| PRJEB42440 | AI2647 | *Klebsiella pneumoniae* | MAD03 | 307 | KPC-3 | 2 | ≤0.06 | ≤0.06 | 8 | 1 | 64 | 0.5 | >64 | 2 | 64 | ≤0.06 |
| PRJEB42440 | AI2648 | *Klebsiella pneumoniae* | MAD03 | 307 | KPC-3 | 2 | ≤0.06 | ≤0.06 | 8 | 0.5 | >64 | 2 | >64 | 2 | >64 | 0.125 |
| PRJEB42440 | AI2649 | *Klebsiella pneumoniae* | MAD03 | 307 | KPC-3 | 2 | ≤0.06 | ≤0.06 | 2 | 0.125 | >64 | 1 | >64 | 1 | >64 | ≤0.06 |
| PRJEB42440 | AI2650 | *Klebsiella pneumoniae* | MAD03 | 307 | KPC-3 | ≤0.06 | ≤0.06 | ≤0.06 | 1 | 0.125 | 64 | 0.25 | >64 | 1 | >64 | 0.125 |
| PRJEB42440 | AI2651 | *Klebsiella pneumoniae* | MAD03 | 459 | KPC-3 | 8 | ≤0.06 | ≤0.06 | 8 | 0.5 | 32 | 0.25 | >64 | 2 | >64 | 0.25 |
| PRJEB42440 | AI2652 | *Klebsiella pneumoniae* | MAD03 | 307 | KPC-3 | 2 | ≤0.06 | ≤0.06 | 4 | 2 | 32 | 0.25 | >64 | 4 | >64 | 0.125 |
| PRJEB42440 | AI2653 | *Klebsiella pneumoniae* | MAD03 | 307 | KPC-3 | 4 | ≤0.06 | ≤0.06 | 4 | 0.125 | 32 | 0.25 | 16 | 1 | >64 | 0.125 |
| PRJEB42440 | AI2901 | *Klebsiella pneumoniae* | MAD03 | 307 | KPC-3 | 8 | ≤0.06 | ≤0.06 | 16 | 1 | 32 | ≤0.06 | >64 | 2 | 64 | ≤0.06 |
| PRJEB42440 | AI2654 | *Klebsiella pneumoniae* | MAD03 | 307 | KPC-3 | 2 | ≤0.06 | ≤0.06 | 8 | 1 | 32 | 1 | 64 | 1 | >64 | 0.125 |
| PRJEB42440 | AI2655 | *Klebsiella pneumoniae* | MAD03 | 307 | KPC-3 | 2 | ≤0.06 | ≤0.06 | 4 | 0.5 | 32 | 0.25 | >64 | 1 | >64 | 0.125 |
| PRJEB42440 | AI2940 | *Klebsiella pneumoniae* | BAL01 | 258 | KPC-3 | 32 | ≤0.06 | ≤0.06 | 16 | 0.5 | >64 | 1 | >64 | 4 | >64 | 0.25 |
| PRJEB42440 | AI2805 | *Klebsiella pneumoniae* | AND04 | 512 | KPC-3 | >64 | ≤0.06 | 1 | 32 | 0.125 | >64 | 2 | >64 | 32 | >64 | 0.25 |
| PRJEB42440 | AI2806 | *Klebsiella pneumoniae* | AND04 | 512 | KPC-3 | 64 | ≤0.06 | 1 | 16 | 0.125 | >64 | 1 | >64 | 8 | >64 | 0.25 |
| PRJEB42440 | AI2807 | *Klebsiella pneumoniae* | AND04 | 512 | KPC-3 | >64 | ≤0.06 | 1 | 32 | 0.125 | >64 | 2 | >64 | 8 | >64 | 0.125 |
| PRJEB42440 | AI2808 | *Klebsiella pneumoniae* | AND04 | 512 | KPC-3 | 64 | ≤0.06 | 0.5 | 16 | ≤0.06 | >64 | 4 | >64 | 8 | >64 | 0.25 |
| PRJEB42440 | AI2809 | *Klebsiella pneumoniae* | AND04 | 512 | KPC-3 | 64 | ≤0.06 | 0.5 | 8 | 0.5 | >64 | 2 | >64 | 4 | >64 | ≤0.06 |
| PRJEB42440 | AI2810 | *Klebsiella pneumoniae* | AND04 | 512 | KPC-3 | >64 | ≤0.06 | 0.5 | 32 | 0.125 | >64 | 4 | 16 | 16 | >64 | 0.25 |
| PRJEB42440 | AI2811 | *Klebsiella pneumoniae* | AND04 | 512 | KPC-3 | >64 | ≤0.06 | 0.5 | 8 | 0.25 | >64 | 2 | >64 | 1 | >64 | 0.25 |
| PRJEB42440 | AI2812 | *Klebsiella pneumoniae* | AND04 | 512 | KPC-3 | >64 | ≤0.06 | 0.5 | 32 | 0.125 | >64 | 4 | >64 | 8 | >64 | 0.25 |
| PRJEB42440 | AI2813 | *Klebsiella pneumoniae* | AND04 | 512 | KPC-3 | 64 | ≤0.06 | 0.25 | 64 | 0.5 | >64 | 2 | >64 | 32 | >64 | 0.25 |
| PRJEB42440 | AI2814 | *Klebsiella pneumoniae* | AND04 | 512 | KPC-3 | >64 | ≤0.06 | 0.25 | 32 | 0.125 | >64 | 2 | >64 | 8 | >64 | 0.125 |
| PRJEB42440 | AI2815 | *Klebsiella pneumoniae* | AND04 | 512 | KPC-3 | >64 | ≤0.06 | 0.25 | 32 | 0.125 | >64 | 2 | >64 | 8 | >64 | 0.125 |
| PRJEB42440 | AI2816 | *Klebsiella pneumoniae* | AND04 | 512 | KPC-3 | >64 | ≤0.06 | 1 | 32 | 0.125 | >64 | 4 | >64 | 8 | >64 | 0.125 |
| PRJEB42440 | AI2817 | *Klebsiella pneumoniae* | AND04 | 512 | KPC-3 | >64 | ≤0.06 | 1 | 16 | 0.125 | >64 | 2 | >64 | 8 | >64 | 0.25 |
| PRJEB53700 | 20200684 | *Citrobacter freundii* | AND17 | 18 | KPC-3 | 4 | ≤0.06 | ≤0.06 | 4 | 0.125 | 64 | 0.125 | >64 | 4 | >64 | 0.125 |
| PRJEB53700 | 20200614 | *Klebsiella pneumoniae* | AND01 | 307 | KPC-3 | 16 | ≤0.06 | ≤0.06 | 8 | 0.125 | 64 | 0.5 | >64 | 2 | >64 | 0.125 |
| PRJEB53700 | 20220707 | *Klebsiella pneumoniae* | AND07 | 258 | KPC-3 | 16 | ≤0.06 | ≤0.06 | 8 | 0.25 | 64 | 0.25 | >64 | 2 | >64 | ≤0.06 |
| PRJEB53700 | 20220496 | *Klebsiella pneumoniae* | AND14 | 35 | KPC-3 | 8 | ≤0.06 | ≤0.06 | 8 | ≤0.06 | 32 | ≤0.06 | >64 | 2 | >64 | 0.125 |
| PRJEB53700 | 20220292 | *Klebsiella pneumoniae* | AND14 | 512 | KPC-3 | >64 | 0.25 | 32 | >64 | 1 | >64 | 1 | >64 | 16 | >64 | 0.5 |
| PRJNA1133624 | ARGA00247 | *Klebsiella pneumoniae* | GAL02 | 512 | KPC-3 | 16 | ≤0.06 | ≤0.06 | 16 | 0.125 | >64 | 0.125 | >64 | 4 | >64 | 0.125 |
| PRJNA1133624 | ARGA00269 | *Klebsiella pneumoniae* | GAL02 | 512 | KPC-3 | 16 | ≤0.06 | ≤0.06 | 16 | 1 | >64 | 0.5 | >64 | 2 | >64 | 0.125 |
| PRJNA1133624 | ARGA00346 | *Klebsiella pneumoniae* | GAL02 | 512 | KPC-3 | >64 | ≤0.06 | 1 | 64 | 0.125 | >64 | ≤0.06 | >64 | 4 | >64 | 0.125 |
| PRJEB42440 | AI2706 | *Klebsiella pneumoniae* | GAL02 | 512 | KPC-3 | >64 | ≤0.06 | 1 | 32 | 0.125 | >64 | 2 | >64 | 8 | >64 | 0.25 |
| PRJEB42440 | AI2707 | *Klebsiella pneumoniae* | GAL02 | 512 | KPC-3 | 64 | ≤0.06 | 0.5 | 32 | 0.125 | >64 | 0.5 | >64 | 4 | >64 | 0.125 |
| PRJEB42440 | AI2709 | *Klebsiella pneumoniae* | GAL02 | 678 | KPC-3 | 8 | ≤0.06 | ≤0.06 | 8 | 0.125 | 64 | ≤0.06 | >64 | 8 | >64 | 0.125 |
| PRJEB42440 | AI2714 | *Klebsiella pneumoniae* | GAL02 | 512 | KPC-3 | 64 | ≤0.06 | 0.25 | 32 | 0.125 | >64 | 1 | >64 | 2 | >64 | 0.125 |
| PRJEB42440 | AI2716 | *Escherichia coli* | GAL02 | 131 | KPC-3 | ≤0.06 | ≤0.06 | ≤0.06 | 0.125 | 0.125 | ≤0.06 | ≤0.06 | 0.25 | 0.125 | ≤0.06 | ≤0.06 |
| PRJEB42440 | AI2722 | *Klebsiella pneumoniae* | GAL02 | 307 | KPC-3 | 4 | ≤0.06 | ≤0.06 | 8 | 0.5 | >64 | 0.125 | >64 | 1 | >64 | ≤0.06 |
| PRJEB42440 | AI2723 | *Klebsiella pneumoniae* | GAL02 | 258 | KPC-3 | 16 | ≤0.06 | ≤0.06 | 8 | ≤0.06 | 64 | 0.125 | >64 | 2 | >64 | 0.125 |
| PRJEB53700 | 20220166 | *Klebsiella pneumoniae* | AND14 | 512 | KPC-23 | 4 | ≤0.06 | ≤0.06 | 4 | ≤0.06 | 32 | 0.125 | >64 | 16 | >64 | 0.25 |
| PRJEB53700 | 20211109 | *Klebsiella pneumoniae* | AND03 | 512 | KPC-31 | 2 | ≤0.06 | 1 | 0.125 | 0.125 | 64 | 8 | >64 | >64 | 16 | 0.5 |
| PRJEB53700 | 20220294 | *Klebsiella pneumoniae* | AND14 | 512 | KPC-66 | 4 | 0.25 | 1 | 0.25 | 0.25 | 64 | 1 | >64 | 32 | 4 | 0.125 |
| PRJEB53700 | 20220366 | *Klebsiella pneumoniae* | AND01 | 512 | KPC-132 | 16 | ≤0.06 | 2 | 4 | 0.25 | >64 | 4 | >64 | >64 | >64 | 0.25 |
| MEM: meropenem; M/X: meropenem/xeruborbactam; M/V: meropenem/vaborbactam; IMP: imipenem; I/R: imipenem/relebactam; FEP: cefepime; F/T: cefepime/taniborbactam; CAZ: ceftazidime; C/A: ceftazidime/avibactam; ATM: aztreonam; A/A: aztreonam/avibactam.  ^a^ EUCAST breakpoints indicated for Enterobacterales. Clinical breakpoints for combinations that have not yet approved (meropenem/xeruborbactam and cefepime/taniborbactam) were interpreted using those of the β-lactam partner. Avibactam, relebactam, and taniborbactam were tested at a fixed concentration of 4 mg/L, while vaborbactam and xeruborbactam were tested at 8 mg/L.  ^b^ N/A: not available | | | | | | | | | | | | | | | | |

| **Table S5.** Antimicrobial susceptibility data for meropenem/xeruborbactam compared to other newly developed β-lactamase inhibitor/β-lactam combinations against MBL-producing Enterobacterales (n=100). | | | | | | | | | | | | | | | | |
| --- | --- | --- | --- | --- | --- | --- | --- | --- | --- | --- | --- | --- | --- | --- | --- | --- |
| **BioProject ID** | **Strain number** | **Species** | **Hospital code** | **MLST^b^** | **Carbapenemase** | **MIC (mg/L)^a^** | | | | | | | | | | |
|  |  |  |  |  |  | **MEM**  **(R>8)** | **M/X**  **(R>8)** | **M/V**  **(R>8)** | **IMP**  **(R>4)** | **I/R**  **(R>2)** | **FEP**  **(R>4)** | **F/T**  **(R>4)** | **CAZ**  **(R>4)** | **C/A**  **(R>8)** | **ATM**  **(R>4)** | **A/A**  **(R>4)** |
| PRJEB53700 | 20210672 | *Enterobacter cloacae* complex | AND03 | 96 | IMP-22 | 2 | ≤0.06 | 2 | 2 | 1 | 8 | 8 | >64 | >64 | 0.5 | 0.125 |
| PRJEB42440 | AI2992 | *Enterobacter cloacae* complex | NAV01 | 96 | IMP-8 | 0.25 | ≤0.06 | 0.25 | 0.5 | 0.5 | 16 | 16 | >64 | >64 | >64 | 1 |
| PRJEB53686 | 20190123 | *Klebsiella pneumoniae* | AND07 | 1873 | IMP-8 | 0.5 | ≤0.06 | 0.5 | 0.5 | 0.5 | 8 | 8 | >64 | >64 | ≤0.06 | ≤0.06 |
| PRJEB53700 | 20190018 | *Klebsiella oxytoca* | AND05 | 2 | IMP-8 | 0.5 | ≤0.06 | 0.5 | 2 | 2 | 16 | 32 | >64 | >64 | >64 | 0.125 |
| PRJEB53686 | 20190067 | *Klebsiella pneumoniae* | AND08 | 464 | IMP-8 | 1 | ≤0.06 | 1 | 1 | 1 | >64 | 32 | >64 | >64 | >64 | 0.25 |
| PRJEB39112 | AI2830 | *Klebsiella pneumoniae* | GAL03 | 147 | NDM-1 | 4 | ≤0.06 | 2 | 8 | 8 | 16 | 1 | >64 | >64 | 64 | 2 |
| PRJEB42440 | AI2880 | *Klebsiella pneumoniae* | MAD03 | 101 | NDM-1 | 8 | ≤0.06 | 8 | 8 | 8 | >64 | 8 | >64 | >64 | >64 | 0.25 |
| PRJEB39112 | AI2898 | *Klebsiella pneumoniae* | MAD03 | 101 | NDM-1 | 4 | ≤0.06 | 2 | 2 | 2 | >64 | 2 | >64 | >64 | >64 | 0.125 |
| PRJEB39112 | AI3007 | *Klebsiella pneumoniae* | VAL01 | 101 | NDM-1 | 4 | ≤0.06 | 4 | 2 | 2 | 32 | 4 | >64 | >64 | >64 | 0.125 |
| PRJEB42440 | AI2793 | *Klebsiella pneumoniae* | VAL01 | 437 | NDM-1 | 4 | ≤0.06 | 4 | 2 | 2 | 16 | 8 | >64 | >64 | 64 | ≤0.06 |
| PRJEB39112 | AI2934 | *Klebsiella pneumoniae* | AND02 | 395 | NDM-1 | 8 | 0.125 | 8 | 16 | 16 | >64 | 2 | >64 | >64 | >64 | 0.125 |
| PRJEB53686 | 20190525 | *Klebsiella pneumoniae* | AND10 | 147 | NDM-1 | 8 | ≤0.06 | 8 | 4 | 4 | 64 | 1 | >64 | >64 | >64 | 0.5 |
| PRJEB53700 | 20220763 | *Escherichia coli* | AND18 | 12279 | NDM-1 | 1 | ≤0.06 | 1 | 1 | 1 | 64 | 0.5 | >64 | >64 | 64 | ≤0.06 |
| PRJEB53700 | 20220548 | *Escherichia coli* | AND11 | 405 | NDM-1 | 16 | ≤0.06 | 16 | 4 | 4 | >64 | 32 | >64 | >64 | >64 | 4 |
| PRJEB53700 | 20200044 | *Enterobacter cloacae* complex | AND01 | 1397 | NDM-1 | 1 | ≤0.06 | 1 | 2 | 2 | 32 | 0.125 | >64 | >64 | 64 | ≤0.06 |
| PRJEB53700 | 20210743 | *Escherichia coli* | AND06 | 162 | NDM-1 | 1 | ≤0.06 | 0.5 | 0.5 | 0.5 | 32 | 0.5 | >64 | >64 | 32 | ≤0.06 |
| PRJNA1133624 | ARGA00078 | *Klebsiella pneumoniae* | GAL02 | 147 | NDM-1 | 64 | 2 | 64 | 32 | 32 | >64 | 8 | >64 | >64 | >64 | 0.5 |
| PRJNA1133624 | ARGA00152 | *Klebsiella pneumoniae* | GAL02 | 6849 | NDM-1 | 4 | ≤0.06 | 4 | 4 | 2 | 64 | 0.5 | >64 | >64 | >64 | 0.125 |
| PRJNA1133624 | ARGA00195 | *Klebsiella aerogenes* | GAL02 | 93 | NDM-1 | 8 | 0.125 | 8 | 4 | 4 | 32 | 0.5 | >64 | >64 | 0.125 | ≤0.06 |
| PRJNA1133624 | ARGA00205 | *Klebsiella pneumoniae* | GAL02 | 17 | NDM-1 | 4 | ≤0.06 | 4 | 4 | 4 | 64 | 0.5 | >64 | >64 | ≤0.06 | ≤0.06 |
| PRJNA1133624 | ARGA00265 | *Klebsiella pneumoniae* | GAL02 | 147 | NDM-1 | 4 | ≤0.06 | 2 | 4 | 4 | 64 | 0.5 | >64 | >64 | >64 | ≤0.06 |
| PRJNA1133624 | ARGA00373 | *Providencia rettgeri* | GAL02 | 4 | NDM-1 | 4 | 0.125 | 2 | 16 | 16 | 1 | 1 | >64 | >64 | ≤0.06 | ≤0.06 |
| PRJEB53700 | 20200186 | *Enterobacter cloacae* complex | AND08 | 523 | NDM-1 | 16 | 0.5 | 32 | 16 | 8 | >64 | 16 | >64 | >64 | 16 | 1 |
| PRJEB53686 | 20200043 | *Klebsiella pneumoniae* | AND01 | 716 | NDM-1 | 4 | ≤0.06 | 4 | 2 | 2 | >64 | 1 | >64 | >64 | >64 | ≤0.06 |
| PRJEB53700 | 20210174 | *Enterobacter cloacae* complex | AND18 | 742 | NDM-1 | 8 | ≤0.06 | 8 | 4 | 2 | >64 | 4 | >64 | >64 | >64 | 0.5 |
| PRJNA1216752 | I399 | *Klebsiella pneumoniae* | ICA01 | 147 | NDM-14 | >64 | 4 | >64 | 64 | 64 | >64 | 32 | >64 | >64 | >64 | 0.125 |
| PRJEB42440 | AI2779 | *Klebsiella pneumoniae* | VAL01 | 437 | NDM-23 | 2 | ≤0.06 | 2 | 2 | 2 | 32 | 1 | >64 | >64 | 64 | ≤0.06 |
| PRJEB42440 | AI2843 | *Escherichia coli* | CAT01 | 410 | NDM-5 | 4 | ≤0.06 | 4 | 8 | 8 | >64 | 16 | >64 | >64 | >64 | 1 |
| PRJEB53700 | 20220823 | *Klebsiella pneumoniae* | AND07 | 15 | NDM-5 | 4 | ≤0.06 | 4 | 4 | 4 | 64 | 1 | >64 | >64 | >64 | ≤0.06 |
| PRJEB53700 | 20220085 | *Escherichia coli* | AND15 | 167 | NDM-5 | 2 | ≤0.06 | 2 | 4 | 4 | >64 | 4 | >64 | >64 | >64 | 0.25 |
| PRJEB53700 | 20210746 | *Klebsiella pneumoniae* | AND17 | 15 | NDM-5 | 8 | ≤0.06 | 8 | 2 | 2 | 64 | 0.5 | >64 | >64 | 64 | ≤0.06 |
| PRJNA1133624 | ARGA00318 | *Klebsiella pneumoniae* | GAL02 | 11 | NDM-5 | 16 | 0.125 | 16 | 4 | 4 | >64 | 4 | >64 | >64 | >64 | 0.125 |
| PRJNA1133624 | ARGA00391 | *Enterobacter cloacae* complex | GAL02 | 1718 | NDM-5 | 32 | 0.25 | 32 | 4 | 4 | >64 | 4 | >64 | >64 | >64 | ≤0.06 |
| PRJEB42440 | AI2858 | *Escherichia coli* | CAT02 | 648 | NDM-7 | 32 | ≤0.06 | 32 | 16 | 16 | >64 | >64 | >64 | >64 | >64 | 8 |
| PRJEB53700 | 20190050 | *Klebsiella pneumoniae* | AND06 | 11 | NDM-7 | 32 | ≤0.06 | 32 | 8 | 8 | >64 | 2 | >64 | >64 | >64 | 0.25 |
| PRJEB53686 | 20190430 | *Escherichia coli* | AND12 | 10087 | NDM-7 | 8 | ≤0.06 | 8 | 2 | 2 | 32 | 1 | >64 | >64 | ≤0.06 | ≤0.06 |
| PRJEB53686 | 20190856 | *Klebsiella pneumoniae* | AND16 | 307 | NDM-7 | 16 | 0.125 | 16 | 16 | 16 | >64 | 8 | >64 | >64 | >64 | 0.5 |
| PRJEB42440 | AI2991 | *Klebsiella oxytoca* | NAV01 | 36 | VIM-1 | 0.125 | ≤0.06 | 0.125 | 4 | 4 | 2 | ≤0.06 | >64 | 64 | 0.25 | ≤0.06 |
| PRJEB42440 | AI2839 | *Enterobacter cloacae* complex | CAT01 | 413 | VIM-1 | 1 | ≤0.06 | 1 | 1 | 0.5 | >64 | 2 | >64 | >64 | 0.125 | ≤0.06 |
| PRJEB42440 | AI2848 | *Klebsiella pneumoniae* | CAT01 | 1083 | VIM-1 | 0.25 | ≤0.06 | 0.25 | 1 | 1 | 64 | 0.25 | >64 | >64 | 0.125 | ≤0.06 |
| PRJEB42440 | AI2972 | *Enterobacter cloacae* complex | CLM01 | 114 | VIM-1 | 0.25 | ≤0.06 | 0.125 | 8 | 8 | >64 | 0.5 | >64 | >64 | 64 | 0.125 |
| PRJEB42440 | AI2626 | *Klebsiella pneumoniae* | AND03 | 11 | VIM-1 | 0.5 | ≤0.06 | 0.25 | 2 | 0.5 | 32 | 2 | >64 | 64 | 4 | 0.125 |
| PRJEB42440 | AI2772 | *Enterobacter cloacae* complex | CAT03 | 110 | VIM-1 | 0.5 | ≤0.06 | 0.5 | 2 | 2 | >64 | 1 | >64 | >64 | 8 | 0.5 |
| PRJEB42440 | AI2859 | *Enterobacter cloacae* complex | MAD02 | 133 | VIM-1 | 1 | ≤0.06 | 0.5 | 4 | 4 | >64 | 4 | >64 | >64 | 0.25 | ≤0.06 |
| PRJEB42440 | AI2860 | *Escherichia coli* | MAD02 | 602 | VIM-1 | ≤0.06 | ≤0.06 | ≤0.06 | 1 | 1 | 4 | 0.25 | >64 | 64 | 0.125 | ≤0.06 |
| PRJEB42440 | AI2828 | *Klebsiella oxytoca* | ARA01 | 202 | VIM-1 | 0.25 | ≤0.06 | 0.25 | 1 | 1 | >64 | 0.25 | >64 | >64 | 64 | 0.125 |
| PRJEB42440 | AI2885 | *Klebsiella pneumoniae* | MAD03 | 307 | VIM-1 | 0.25 | ≤0.06 | 0.25 | 4 | 2 | 32 | 0.25 | >64 | >64 | >64 | 0.125 |
| PRJEB42440 | AI2660 | *Klebsiella pneumoniae* | BAL01 | 11 | VIM-1 | 0.5 | ≤0.06 | 0.5 | 4 | 4 | >64 | 2 | >64 | >64 | 0.25 | 0.125 |
| PRJEB42440 | AI2797 | *Enterobacter cloacae* complex | CAT04 | 24 | VIM-1 | 1 | ≤0.06 | 1 | 2 | 1 | >64 | 1 | >64 | >64 | 2 | 0.25 |
| PRJEB42440 | AI2798 | *Enterobacter cloacae* complex | CAT04 | 78 | VIM-1 | 1 | ≤0.06 | 1 | 2 | 1 | >64 | 0.5 | >64 | >64 | >64 | 0.25 |
| PRJEB42440 | AI3012 | *Klebsiella oxytoca* | CAT04 | 2 | VIM-1 | 0.5 | ≤0.06 | 0.5 | 0.5 | 0.5 | >64 | 0.5 | >64 | >64 | 4 | 0.125 |
| PRJEB42440 | AI3013 | *Enterobacter cloacae* complex | CAT04 | 764 | VIM-1 | 1 | ≤0.06 | 1 | 1 | 1 | 64 | 1 | >64 | >64 | 4 | 0.125 |
| PRJEB53700 | 20190130 | *Escherichia coli* | AND11 | 216 | VIM-1 | 0.5 | ≤0.06 | 0.5 | 4 | 4 | 64 | 0.25 | 16 | 8 | 8 | ≤0.06 |
| PRJEB53700 | 20200592 | *Klebsiella pneumoniae* | AND08 | 234 | VIM-1 | 2 | ≤0.06 | 2 | 4 | 4 | 64 | 1 | >64 | >64 | 2 | ≤0.06 |
| PRJEB53700 | 20210949 | *Klebsiella pneumoniae* | AND08 | 219 | VIM-1 | 2 | ≤0.06 | 2 | 2 | 2 | 32 | 0.25 | >64 | >64 | >64 | 0.125 |
| PRJEB53700 | 20220579 | *Escherichia coli* | AND08 | 1193 | VIM-1 | 0.25 | ≤0.06 | 0.25 | 0.25 | 0.125 | 8 | 0.25 | >64 | >64 | 4 | 0.125 |
| PRJEB53700 | 20220343 | *Escherichia coli* | AND08 | 69 | VIM-1 | 0.5 | ≤0.06 | 0.5 | 1 | 1 | 32 | 0.5 | >64 | >64 | 32 | ≤0.06 |
| PRJEB53700 | 20210320 | *Escherichia coli* | AND18 | 744 | VIM-1 | 0.5 | ≤0.06 | 0.5 | 1 | 1 | 32 | 0.5 | >64 | >64 | 0.125 | ≤0.06 |
| PRJEB53700 | 20200150 | *Escherichia coli* | AND02 | 10 | VIM-1 | ≤0.06 | ≤0.06 | ≤0.06 | 0.5 | 0.5 | 2 | ≤0.06 | 64 | 32 | ≤0.06 | ≤0.06 |
| PRJNA1133624 | ARGA00135 | *Raoultella planticola* | GAL02 | N/A | VIM-1 | 32 | 2 | 32 | 32 | 32 | >64 | 64 | 64 | 64 | 1 | 0.5 |
| PRJNA1133624 | ARGA00197 | *Escherichia coli* | GAL02 | 10 | VIM-1 | 0.125 | ≤0.06 | 0.125 | 0.5 | 0.5 | 8 | 0.125 | >64 | >64 | ≤0.06 | ≤0.06 |
| PRJNA1133624 | ARGA00198 | *Enterobacter cloacae* complex | GAL02 | 3297 | VIM-1 | 0.25 | ≤0.06 | 0.125 | 0.5 | 0.5 | >64 | 0.5 | >64 | >64 | >64 | 0.5 |
| PRJNA1133624 | ARGA00245 | *Enterobacter cloacae* complex | GAL02 | 102 | VIM-1 | 4 | ≤0.06 | 2 | 2 | 1 | >64 | 16 | >64 | >64 | >64 | 1 |
| PRJNA1133624 | ARGA00259 | *Enterobacter cloacae* complex | GAL02 | 102 | VIM-1 | 8 | 0.25 | 8 | 4 | 4 | >64 | 2 | >64 | >64 | >64 | 0.25 |
| PRJNA1133624 | ARGA00270 | *Enterobacter cloacae* complex | GAL02 | 102 | VIM-1 | 1 | ≤0.06 | 1 | 1 | 0.5 | >64 | 2 | >64 | >64 | >64 | 0.5 |
| PRJEB53700 | 20220856 | *Klebsiella pneumoniae* | AND11 | 11 | VIM-1 | 0.5 | ≤0.06 | 0.5 | 2 | 0.5 | 32 | 1 | >64 | >64 | 4 | 0.25 |
| PRJEB53700 | 20220459 | *Klebsiella pneumoniae* | AND02 | 15 | VIM-1 | 0.5 | ≤0.06 | 0.5 | 1 | 0.5 | 64 | 0.25 | >64 | >64 | >64 | ≤0.06 |
| PRJEB53700 | 20210875 | *Klebsiella pneumoniae* | AND08 | 20 | VIM-1 | 0.5 | ≤0.06 | 0.5 | 1 | 1 | 16 | 0.25 | >64 | >64 | 4 | ≤0.06 |
| PRJEB53700 | 20220344 | *Citrobacter freundii* | AND17 | 22 | VIM-1 | 0.25 | ≤0.06 | 0.25 | 0.5 | 0.5 | 16 | 0.25 | >64 | >64 | >64 | 0.125 |
| PRJEB53700 | 20190530 | *Klebsiella pneumoniae* | AND08 | 25 | VIM-1 | 0.5 | ≤0.06 | 0.5 | 1 | 1 | 32 | 1 | >64 | >64 | 4 | ≤0.06 |
| PRJEB53700 | 20210679 | *Klebsiella pneumoniae* | AND08 | 35 | VIM-1 | 8 | 1 | 8 | 16 | 16 | 64 | 4 | >64 | >64 | 8 | 0.125 |
| PRJEB53700 | 20210479 | *Klebsiella pneumoniae* | AND08 | 37 | VIM-1 | 0.25 | ≤0.06 | 0.25 | 4 | 2 | 16 | 0.25 | >64 | >64 | 4 | 0.125 |
| PRJEB53700 | 20220649 | *Klebsiella pneumoniae* | AND11 | 39 | VIM-1 | 0.125 | ≤0.06 | ≤0.06 | 0.5 | 0.5 | 4 | 0.125 | 64 | 32 | ≤0.06 | ≤0.06 |
| PRJEB53700 | 20220566 | *Enterobacter cloacae* complex | AND11 | 63 | VIM-1 | 0.5 | ≤0.06 | 0.5 | 0.25 | 0.25 | 64 | 64 | >64 | >64 | 32 | 0.5 |
| PRJEB53700 | 20200366 | *Klebsiella pneumoniae* | AND08 | 70 | VIM-1 | 0.5 | ≤0.06 | 0.5 | 2 | 1 | 16 | 0.25 | >64 | >64 | 4 | ≤0.06 |
| PRJEB53686 | 20200160 | *Enterobacter cloacae* complex | AND17 | 78 | VIM-1 | 0.5 | ≤0.06 | 0.5 | 0.5 | 0.25 | 32 | 0.5 | >64 | >64 | >64 | 0.5 |
| PRJEB53700 | 20190015 | *Enterobacter cloacae* complex | AND05 | 88 | VIM-1 | 1 | ≤0.06 | 1 | 1 | 1 | 32 | 1 | >64 | >64 | 64 | 0.5 |
| PRJEB53700 | 20210276 | *Enterobacter cloacae* complex | AND17 | 90 | VIM-1 | 1 | ≤0.06 | 1 | 2 | 1 | 32 | 0.5 | >64 | >64 | >64 | 0.25 |
| PRJEB53700 | 20220037 | *Klebsiella aerogenes* | AND11 | 93 | VIM-1 | 0.25 | ≤0.06 | 0.25 | 2 | 2 | 16 | 0.125 | >64 | >64 | ≤0.06 | ≤0.06 |
| PRJEB53700 | 20200046 | *Enterobacter cloacae* complex | AND01 | 96 | VIM-1 | 1 | ≤0.06 | 0.5 | 1 | 0.5 | 16 | 0.25 | >64 | >64 | 1 | 0.125 |
| PRJEB53686 | 20190928 | *Citrobacter freundii* | AND08 | 98 | VIM-1 | 1 | ≤0.06 | 1 | 4 | 2 | >64 | 2 | >64 | >64 | 32 | 0.25 |
| PRJEB53700 | 20220446 | *Enterobacter cloacae* complex | AND17 | 102 | VIM-1 | 0.5 | ≤0.06 | 0.5 | 0.25 | 0.25 | >64 | 4 | >64 | >64 | 32 | 0.5 |
| PRJEB53700 | 20220133 | *Enterobacter cloacae* complex | AND15 | 106 | VIM-1 | 1 | ≤0.06 | 1 | 0.5 | 0.5 | 32 | 0.25 | >64 | >64 | 64 | 0.5 |
| PRJEB53700 | 20210405 | *Citrobacter freundii* | AND08 | 111 | VIM-1 | 0.5 | ≤0.06 | 0.5 | 1 | 1 | 32 | 0.5 | >64 | >64 | 16 | 0.125 |
| PRJEB53686 | 20200008 | *Citrobacter freundii* | AND07 | 116 | VIM-1 | 1 | ≤0.06 | 0.5 | 2 | 1 | 32 | 0.25 | >64 | >64 | 1 | ≤0.06 |
| PRJEB53700 | 20200387 | *Klebsiella oxytoca* | AND08 | 135 | VIM-1 | 1 | ≤0.06 | 1 | 4 | 2 | 32 | 0.25 | >64 | >64 | 8 | 0.25 |
| PRJEB53700 | 20220277 | *Klebsiella oxytoca* | AND08 | 145 | VIM-1 | 8 | 0.25 | 4 | 8 | 8 | >64 | 8 | >64 | >64 | 8 | ≤0.06 |
| PRJEB53700 | 20190836 | *Enterobacter cloacae* complex | AND08 | 171 | VIM-1 | 0.5 | ≤0.06 | 0.25 | 1 | 1 | >64 | 1 | >64 | >64 | >64 | 0.25 |
| PRJEB53700 | 20220100 | *Enterobacter cloacae* complex | AND18 | 175 | VIM-1 | 2 | ≤0.06 | 2 | 4 | 2 | >64 | 2 | >64 | >64 | 64 | 2 |
| PRJEB53700 | 20211053 | *Klebsiella oxytoca* | AND08 | 190 | VIM-1 | 2 | ≤0.06 | 2 | 2 | 2 | 64 | 0.5 | >64 | >64 | 64 | 0.5 |
| PRJNA1133624 | 20210354 | *Klebsiella oxytoca* | AND08 | 202 | VIM-1 | 0.5 | ≤0.06 | 0.5 | 2 | 2 | 32 | 0.25 | >64 | >64 | 16 | 0.125 |
| PRJEB53700 | 20211121 | *Klebsiella oxytoca* | AND17 | 226 | VIM-1 | 0.25 | ≤0.06 | 0.25 | 2 | 2 | 32 | 0.25 | >64 | >64 | 4 | ≤0.06 |
| PRJEB53700 | 20190024 | *Klebsiella oxytoca* | AND02 | 237 | VIM-1 | 1 | ≤0.06 | 1 | 4 | 4 | 32 | 0.25 | >64 | >64 | 2 | 0.125 |
| PRJEB53700 | 20210562 | *Klebsiella pneumoniae* | AND08 | 307 | VIM-1 | 0.5 | ≤0.06 | 0.5 | 1 | 1 | 16 | 0.25 | >64 | >64 | 4 | ≤0.06 |
| PRJEB53700 | 20210683 | *Enterobacter cloacae* complex | AND17 | 311 | VIM-1 | 0.25 | ≤0.06 | 0.25 | 1 | 0.5 | 64 | 0.25 | >64 | >64 | >64 | 0.125 |
| PRJEB53700 | 20190241 | *Enterobacter cloacae* complex | AND08 | 344 | VIM-1 | 0.25 | ≤0.06 | 0.25 | 2 | 1 | >64 | 2 | >64 | >64 | 8 | 0.25 |
| PRJEB53686 | 20190025 | *Citrobacter freundii* | AND07 | 432 | VIM-1 | 1 | ≤0.06 | 0.5 | 1 | 1 | 64 | 1 | >64 | >64 | >64 | 0.125 |
| PRJEB53700 | 20210026 | *Klebsiella pneumoniae* | AND17 | 469 | VIM-1 | 8 | 0.25 | 8 | 4 | 4 | >64 | 4 | >64 | >64 | >64 | 0.125 |
| PRJEB53700 | 20210070 | *Klebsiella pneumoniae* | AND18 | 1805 | VIM-33 | >64 | 4 | >64 | 64 | 64 | >64 | 1 | >64 | >64 | 0.25 | 0.125 |
| PRJEB53700 | 20211129 | *Enterobacter cloacae* complex | AND17 | 1381 | VIM-4 | 1 | ≤0.06 | 1 | 2 | 2 | 2 | 0.5 | 16 | 16 | 1 | 0.125 |
| MEM: meropenem; M/X: meropenem/xeruborbactam; M/V: meropenem/vaborbactam; IMP: imipenem; I/R: imipenem/relebactam; FEP: cefepime; F/T: cefepime/taniborbactam; CAZ: ceftazidime; C/A: ceftazidime/avibactam; ATM: aztreonam; A/A: aztreonam/avibactam.  ^a^ EUCAST breakpoints indicated for Enterobacterales. Clinical breakpoints for combinations that have not yet approved (meropenem/xeruborbactam and cefepime/taniborbactam) were interpreted using those of the β-lactam partner. Avibactam, relebactam, and taniborbactam were tested at a fixed concentration of 4 mg/L, while vaborbactam and xeruborbactam were tested at 8 mg/L.  ^b^ N/A: not available | | | | | | | | | | | | | | | | |
